# Two types of somatostatin-expressing GABAergic interneurons in the superficial layers of the mouse cingulate cortex

**DOI:** 10.1371/journal.pone.0200567

**Published:** 2018-07-12

**Authors:** Therese Riedemann, Tobias Straub, Bernd Sutor

**Affiliations:** 1 Ludwig-Maximilians-University, Biomedical Center, Physiological Genomics, Munich, Germany; 2 Ludwig-Maximilians-University, Biomedical Center, Core Facility Bioinformatics, Munich, Germany; University of North Dakota, UNITED STATES

## Abstract

Somatostatin-expressing (SOM^+^), inhibitory interneurons represent a heterogeneous group of cells and given their remarkable diversity, classification of SOM^+^ interneurons remains a challenging task. Electrophysiological, morphological and neurochemical classes of SOM^+^ interneurons have been proposed in the past but it remains unclear as to what extent these classes are congruent. We performed whole-cell patch-clamp recordings from 127 GFP-labeled SOM^+^ interneurons ('GIN') of the superficial cingulate cortex with subsequent biocytin-filling and immunocytochemical labeling. Principal component analysis followed by k-means clustering predicted two putative subtypes of SOM^+^ interneurons, which we designated as group I and group II GIN. A key finding of our study is the fact that these electrophysiologically and morphologically distinct groups of SOM^+^ interneurons can be correlated with two neurochemical subtypes of SOM^+^ interneurons described recently in our laboratory. In particular, all SOM^+^ interneurons expressing calbindin but no calretinin could be classified as group I GIN, whereas all but one neuropeptide Y- and calretinin-positive interneurons were found in group II.

## Introduction

Within the cerebral cortex, inhibitory modulation of excitatory information processing is crucial for proper network signaling. GABAergic interneurons represent around 10–20% of all neurons within the cerebral cortex. Of these, somatostatin-expressing interneurons represent around one third of all GABAergic interneurons [1,2]. The cingulate cortex is a brain region that seems particularly vulnerable to dysfunctions of GABAergic signaling and many neurological diseases are accompanied by a specific loss of somatostatin-expressing (SOM^+^) interneurons within this brain area [3–5]. In order to understand why a certain neurological disease is accompanied by a specific loss of one type of GABAergic interneurons, the specific properties and functions of these interneurons need to be determined. Efforts to classify GABAergic interneurons are faced with their enormous diversity in terms of morphology, neurochemistry and physiology. The great variability of their properties initiated an extensive categorization of these cells, the main criteria being single cell morphology, expression of calcium-binding proteins and/or neuropeptides and the pattern of action potential discharge induced by depolarizing current pulses [6–13]. Given that different studies have used diverging classification parameters, it remains unclear as to what extent different classification schemes are congruent or at least comparable. Ideally, an interneuron classification would not only be based on electrophysiological and morphological but also on molecular data and consider as many well-defined variables as possible in order to paint a comprehensive picture of a certain interneuron type (see e.g. [11,14]). Recently, interneuron classification has made substantial progress by single-cell transcriptomics [15–18]. However, detailed electrophysiological analysis of neurons providing sufficient information for a meaningful cluster analysis requires long-lasting recordings. This is incompatible with high-quality single-cell transcriptomic analysis [16]. In a previous study, we described distinct neurochemical subtypes of SOM^+^ interneurons within the cingulate cortex of the so-called GIN mouse line (FVB-Tg(GadGFP)45704Swn/J;[2,19]). Similar to other cortical regions [20–22], SOM^+^ interneurons of the cingulate cortex (CC) exhibited a great heterogeneity in terms of calretinin (CR), calbindin (CB) and neuropeptide Y (NPY) expression. In the present study, we analyzed the electrophysiological and morphological properties of SOM^+^ interneurons in this brain region and, particularly, we investigated the question whether these properties overlap with neurochemical subgroups of SOM^+^ interneurons described previously in our laboratory. We were able to quantitatively characterize two distinct electrophysiological and morphological groups of SOM^+^ interneurons by performing a principal component analysis followed by k-means clustering. These two subgroups, designated as group I and group II GIN, differed not only in their passive and active membrane properties and in their action potential discharge patterns, but also in their morphological complexity. A key finding of this study is that it was possible to assign the expression of certain neurochemical marker proteins to one of the two SOM^+^ subgroups, promoting our understanding of the neurochemical and functional diversity of SOM^+^ interneurons in the cingulate cortex.

## Materials and methods

### Animals

Experiments were performed on animals of a transgenic mouse line (FVB-Tg(GadGFP)45704Swn/J; [19]), where in a subset of inhibitory interneurons the enhanced green fluorescent protein (eGFP) is expressed under the control of the glutamic acid decarboxylase 67 promotor (GAD67). The age of all mice used ranged between 21 and 54 days. Animals were obtained from Jackson Laboratories (ME, USA) and were bred in the institute’s animal facility that was licensed by the Bavarian State authorities (File number: 5.1-5682/LMU/BMC/CAM). Prior decapitation, animals were deeply anaesthetized by CO_2_ exposure until the extinction of all reflexes. These experiments are, according to the German animal protection law (TierSchG) classified as notifiable. They were approved by the institutional committees on animal care (Core Facility Animal Models / Ludwig-Maximilians-University Munich) and by the Bavarian State authorities, conforming to international guidelines on the ethical use of animals.

### Slice preparation

Preparation of coronal slices (thickness: 300 μm) was performed as previously described [23]. After the cutting procedure, the slices were collected and submerged in artificial cerebro-spinal fluid (ASCF) containing (in mM): NaCl (125), KCl (3), NaH_2_PO_4_ (1.25), NaHCO_3_ (25), CaCl_2_ (2), MgCl_2_ (2), ascorbic acid (0.4) and D-Glucose (25). The ACSF was continuously perfused with 95% O_2_ / 5% CO_2_ in order to maintain a pH of 7.4. The slices were incubated for one hour at 28°C and for another hour at room temperature. For electrophysiological analysis, single slices were transferred to a recording chamber mounted on the stage of an upright microscope (Zeiss Axioskop FS equipped with a 40x objective, 0.75 numerical aperture [NA]). The recording chamber was continuously perfused with ACSF the temperature of which was maintained at 27–28°C with the help of a temperature controller (TC-324B, Warner Instrument Corp., Connecticut, USA).

### Whole-cell recordings

GFP-expressing inhibitory interneurons (GIN) of layers 2 and 3 of the anterior cingulate cortex were visualized and identified by means of an upright microscope equipped with differential-interference-contrast (DIC)-infrared optics and epifluorescence (filter set: Zeiss BP450-490, LP520). Fluorescence and infrared images were acquired with the help of a CCD camera (Orca-ER, Hamamatsu, Shizouka, Japan). The electrodes for whole-cell patch-clamp recordings were fabricated from borosilicate glass capillaries (OD: 1.5 mm, ID: 0.86 mm, Hugo Sachs Elektronik-Harvard Apparatus, March-Hugstetten, Germany) and were filled with a solution containing (in mM): K-gluconate (135), KCl (4), NaCl (2), EGTA (0.2), HEPES (10), Mg-ATP (4), Na-GTP (0.5), and phosphocreatine (10). The solution had an osmolarity of 290 mOsm and a pH of 7.3. Biocytin (0.3–0.5%) was added to the electrode solution and the pipettes were filled by means of the so-called backfilling procedure to avoid high background staining. In some experiments, potassium gluconate was replaced by potassium methylsulfate. The electrodes (resistance: 4–8 MΩ) were connected to the amplifier’s headstage via a chlorided silver wire. A silver / silver chloride–pellet immersed into the recording solution served as reference electrode. Somatic whole-cell recordings were made in current clamp mode using an ELC 03XS amplifier (npi electronics, Tamm, Germany). Bias and offset current were zeroed before giga seal formation. After rupture of the membrane, the electrode capacitance and series resistance were compensated as described by Riedemann et al. [23].

### Analysis of electrophysiological parameters

For the analysis of passive membrane properties (input resistance, membrane time constants, cell input capacitance) twenty hyperpolarizing current steps (ΔI, 5–20 pA, 500 ms) were injected into the cells. The voltage responses were averaged and measurements of the amplitude (ΔV) were taken at the end of the current pulse (i.e. at steady state). Attention was paid so that the current-induced voltage deflection never surpassed 10 mV in amplitude. The input resistance (R_N_) was then calculated from Ohm’s law (R_N_ = ΔV/ΔI). The somatic membrane time constant (τ_0_) and the first equalizing time constant (τ_1_) were obtained by fitting a double-exponential function to the first 25–70 ms of the averaged current-induced voltage response. The membrane input capacitance (C_N_) was determined according to the method described by Zemankovics et al. (2010). The neurons' current-voltage relationships (IV-curves) were obtained by injecting hyper- and depolarizing current steps (1000 ms in duration). The step amplitudes and the step increments (5–20 pA) were adjusted to cover a membrane potential range between about -100 mV and just subthreshold levels. We determined the steady state IV-curve (i.e. measurements were taken at the end of the current pulses) and the IV-curve at a time point, when the voltage response to the largest hyperpolarizing current step reached a maximum (usually around 70–150 ms post current step onset). To obtain the IV-curves, the amplitudes of the voltage responses were plotted as a function of the current intensities injected and the data points were interpolated using the smoothing spline algorithm supplied by IGOR Pro 6 (WaveMetrics, Lake Oswego, USA). The interpolated IV-curves were differentiated resulting in a relationship, where the slope resistance of a cell is displayed as a function of the current injected (so-called R_N_-curve). In order to describe the rectifying properties of the current-voltage relationship, the R_N_-curve was normalized with respect to the steady state slope resistance at resting membrane potential (i.e. input resistance at 0 pA derived from the R_N_-curve). A rectification ratio of 1 for all responses represents a linear current-voltage-relationship, values larger than 1 indicate inward rectification in hyperpolarizing and/or outward rectification in depolarizing direction. Values smaller than 1 indicate outward rectification in hyperpolarizing and/or inward rectification in depolarizing direction. The rectification index (i.e. the relation of the input resistances at any potential to that at resting membrane potential) was determined at a membrane potential of about -100 mV. Most GIN displayed a prominent sag potential that could be blocked by the *I*_H_ current antagonist ZD7288 ([24], data not shown). For the description of this sag potential, a sag index was calculated according to Halabisky et al. [20] using the equation (R_max_-R_sag_)/R_max_.

The properties of single action potentials were derived from recordings in which action potentials were elicited by means of just suprathreshold current steps (50 ms). The spikes were analyzed as follows: the amplitude was taken as the difference between the resting membrane potential and the maximum voltage deflection. Duration, rising slope and spike threshold were determined according to methods given by Bean [25]: Single spikes were differentiated and the spike duration corresponded to the temporal difference between the maximum and the minimum of the differentiated spike. The rising slope was equal to the maximum of the first spike derivative and the rise-to-fall ratio was calculated from the quotient of the maximum and the absolute value of the minimum. For the spike threshold analysis, the differentiated spike was plotted as a function of the membrane voltage. In this phase plane, the threshold corresponded to the point where the rising slope of the membrane voltage displayed a sudden increase. For the analysis of action potential discharge patterns, 30 depolarizing current steps (1000 ms in duration) with increments of usually 5–20 pA were injected. To investigate the properties of the discharge patterns quantitatively, we determined the firing frequencies from the interspike intervals (ISI) and plotted these values as a function of the spike times. These *F*-*t*-relationships were determined for all effective current strengths yielding an array of curves from which the following parameters were derived: (1) ratio of the frequencies of the first and the second ISI (F_ISI1_ and F_ISI2_), (2) ratio of the frequencies of the first and the last ISI (F_ISILast_) (i.e. adaptation index), (3) ratio of the frequency of the first ISI to the mean frequency (F_ISIMean_). All these measurements were performed at current strengths, where we did neither encounter fluctuation driven discharge [26] nor action potential shunting. In addition, we plotted the initial discharge frequency (determined from the first ISI), the frequency of the second ISI, the steady state frequency and the mean frequency as a function of the injected current. In these *F*-*I* diagrams, the data points could be fitted by either a single linear function (meaning that the gain of the neuron was constant for all current strengths analyzed) or successively by two linear functions. In the latter case, we formed a ratio between the slope of the first and the second line. In addition, we determined the spike threshold, the spike duration and the rising slope of the first, the second and the last spike in a train. The spike afterhyperpolarization (AHP) was characterized by determination of the following parameters: AHP time course (monophasic or biphasic), time to negative peak, amplitude, duration and rising slope of the fast initial AHP component of the first, the second and the last spike.

In order to detect spontaneous synaptic activity reaching the soma either directly or via dendrites, we performed current clamp recordings for 5 or 10 minutes (filter: 20 kHz) from the neurons' somata. We preferred current clamp instead of voltage clamp recordings, since not all of the neurons were electrotonically compact. This would introduce an artificial variability due to space clamp problems [23]. Spontaneous synaptic events "seen" by the electrode positioned at the soma were automatically detected using the algorithm provided by the NeuroMatic plugin (version 2.00) for IgorPro. The detection threshold was set to 0.5 mV. Only monophasic synaptic events were analyzed and the following parameters were determined: frequency, peak amplitude, rising slope and duration.

### Data acquisition and analysis

Recorded voltage signals were amplified (x 20), filtered at 20 kHz and digitized at sampling rates of 10 or 20 kHz. Data acquisition and generation of command pulses was accomplished by means of an analogue-digital converter (PCI-6024E, National Instruments, Austin, TX, USA) in conjunction with the CellWorks data acquisition software (npi, Tamm, Germany). Data analysis was performed using IGOR Pro 6 (WaveMetrics, Lake Oswego, USA) together with the NeuroMatic IGOR plugin (version 2.00, www.neuromatic.thinkrandom.com). Graphs were either prepared in IgorPro, in Excel (Microsoft, USA) or in GraphPad Prism (La Jolla, USA).

### Visualization of biocytin-injected neurons and labeling for immunofluorescence

At the end of the recordings, the biocytin-filled patch-pipettes were very carefully withdrawn from the soma of the neuron. The slices were then fixed for 12 hours in phosphate-buffered saline (PBS) containing freshly prepared 4% paraformaldehyde. After fixation, slices were washed at least 6 times with PBS containing 0.3% Triton-X100 and kept in this solution for 48 hours. Neurons were visualized by incubating the slices in Alexa 594- or Alexa 488-coupled streptavidine (diluted 1:1000 in PBS, Molecular Probes, USA) for at least 48 hours. Next, neurons were labeled for immunofluorescence as described by Riedemann et al. (2016a). The following antibodies were used: chicken anti-GFP (Millipore, Billerica, MA, USA, 06–896, dilution: 1:400); rat anti-somatostatin (1:200, Millipore, MAB354, monoclonal), rabbit anti-calretinin (1:1000, SWANT, Bellinzona, Switzerland, 7699/3H, polyclonal), mouse anti-calretinin (1:2000, SWANT, 6B3, monoclonal), mouse anti-calbindin (1:1000, SWANT, 300, monoclonal) and sheep anti-neuropeptide Y (1:1000, Millipore, AB1583, polyclonal). All antibodies were diluted in PBS containing 0.3% Triton and incubated for at least 48 hours. The secondary antibody directed against chicken was Alexa Fluor 488-conjugated goat anti-chicken IgG (Invitrogen, A11039, 1:500). Secondary antibodies directed against mouse were: CF 405S-conjugated goat anti-mouse IgG (Biotium, Hayward, CA, USA, 20082, 1:500), Dylight 405-conjugated goat anti-mouse (Rockland, Limerick, PA, USA, 610-146-002, 1:1000), Alexa Fluor 594-conjugated goat anti-mouse IgG (Molecular Probes, Waltham, MA, USA, A-11032, 1:500), Cy3-conjugated goat anti-mouse IgG (Dianova, Hamburg, Germany, 115-165-003, 1:500), Dylight 649-conjugated goat anti-mouse IgG (Dianova, 115-606-072, 1:500). Secondary antibodies against rabbit were as follows: Cy3-conjugated donkey anti-rabbit (Dianova, 711-165-152, 1:500), Cy5-conjugated goat anti-rabbit IgG (Dianova, 111-175-144; 1:500), Alexa Fluor 647-conjugated goat anti-rabbit IgG (Dianova, 111-605-144, 1:500). Secondary antibodies used against rat included: Cy3-conjugated goat anti-rat IgG (Dianova, 112-165-167, 1:500), Alexa Fluor A647-conjugated goat anti-rat (Molecular Probes, A-21247, 1:500), and Dylight 649-conjugated goat anti-rat IgG (Rockland, 612-143-002, 1:1000). The secondary antibodies directed against sheep were Dylight 549-conjugated rabbit anti-sheep IgG (Rockland, 613-442-002, 1:1000) and Dylight 549-conjugated donkey anti-sheep IgG (Rockland, 613-742-168, 1:1000). Following secondary antibody incubation, slices were washed extensively before being wet-mounted onto glass slides using spacers to avoid compression. Sections were coverslipped with a MOWIOL-based mounting medium [27] tested for autofluorescence.

### Confocal imaging of biocytin-injected neurons

Confocal images were acquired with a LSM710 laser scanning microscope (Zeiss, Jena, Germany) using the ZEN software (Zeiss). The fluorescent signals were elicited and detected in a sequential mode. Alexa 405-conjugated probes/DAPI were excited at 405 nm (diode laser), GFP was visualized at 488 nm (argon laser), Alexa 594/Cy3-conjugated probes at 561 nm (Helium/Neon, He/Ne laser), and Alexa 647-/Dylight649-conjugated probes at 633 nm (He/Ne laser). Images were scanned at a resolution of 2048 x 2048 pixels when testing for expression of marker proteins and at a resolution of 4096 x 4096 pixels when analyzing neuronal morphology. Images were scanned using the following objectives: 10x air (NA = 0.3), 25x water (NA = 0.8), and 40x water (NA = 1.1). For spine analysis, images were scanned at a resolution of 1048 x 1048 pixels with a 63x water immersion objective (NA = 1.2). All fluorescence emission filters were non-overlapping and centered around the emission maximum of the respective secondary antibody signal. Z-stacks through the depth of the sections (30–50 μm) were acquired and the individual images collapsed onto one focal plane (maximum intensity projection) in order to obtain a two-dimensional image. Z-stack intervals ranged from 0.2 μm to 1.12 μm.

### Morphological reconstruction and morphometric analysis of biocytin-injected neurons

Stained neurons were reconstructed either manually or semi-automatically. Reconstructions were performed using the NeuronStudio software (Mount Sinai School of Medicine, USA). Confocal image stacks of biocytin-filled neurons were uploaded into the NeuronStudio software and the correct voxel dimensions were adjusted. Dendrites and axons (partial reconstruction of axons) were traced individually. Sholl analysis of dendritic processes was performed using the NeuronStudio software. Sholl discs were spaced at 10 μm intervals. The following variables were included into the morphometric analysis: total length of the dendritic tree, dendritic tree length at 10, 100, 200 and 300 μm and beyond Sholl radius, origin of dendrite, total number of branching points, number of branching points at 10, 100, 200 and 300 μm and beyond Sholl radius, number of primary dendrites, direction of axon, origin of axon, distance to pia, existence of spines.

### Principal component analysis and cluster analysis

Principal component analysis (PCA) was performed using the 'prcomp' function implemented in R (R-project.org). Categorical variables were transformed into dummy binary ones and all variables were scaled to unit variance prior to analysis. The PCA comprised the analysis of 127 GIN, 6 pyramidal cells and 1 neurogliaform neuron. Only cells with complete electrophysiological and morphological data sets were included in the analysis. We performed k-means clustering on the first four principal components looking for 3 clusters using 25 random start sets and a maximum of 1000 iterations.

### Statistics

Group I and group II GIN were examined by pairwise statistical analysis of the single electrophysiological and morphological parameters obtained. Normal distribution of data points was assessed by D’Agostino and Pearson Omnibus Normality test. In case of a normal distribution of data points, two-tailed students' t-test were performed for statistical comparisons between GIN subtypes (group I GIN: n = 39; group II GIN: n = 88). In case data points were not distributed normally, Mann-Whitney tests were performed. Sholl analyses on GIN subtypes and pyramidal cells were analyzed by two-way Anova with Bonferroni post tests. Significance levels were *p*<0.05; *p*<0.01 and *p*<0.001. Data are presented as mean ± standard deviation (if not stated otherwise). High quality electrophysiological recordings were performed on 132 GIN, of which 127 received biocytin-filling and were successfully reconstructed.

## Results

### Neurochemical subtypes of GIN

As previously reported [2], the so-called "green fluorescent protein (GFP) expressing interneurons" (GIN, [19]) of the mouse cingulate cortex represent a heterogeneous group of cells with respect to their neurochemical profile. In order to investigate whether neurochemically defined subgroups of GIN display distinct electrophysiological and/or morphological features, the expression of calretinin (CR), calbindin (CB) and neuropeptide Y (NPY), respectively, was analyzed in biocytin-labeled and electrophysiologically characterized GIN. Altogether, we investigated the electrophysiological and morphological properties of 127 GIN and 6 pyramidal neurons. Out of these 127 GIN, 98 cells were also subjected to immunolabeling for either CR and/or CB and/or NPY. Forty-seven out of 98 GIN expressed CR alone, 6 CB alone, 31 CR and CB, 8 CR and NPY and 6 CB and NPY. The relative contributions of the various subtypes to the whole sample corresponds to the relative distributions described in our recent study [2]. Thirty-seven out of 38 biocytin-filled GIN were SOM^+^, confirming their somatostatinergic phenotype. The pyramidal cells served as an "internal control", particularly with regard to the principal component analysis and k-means clustering.

### Principal component analysis and k-means clustering to identify distinct interneuron clusters

**Fig 1:**
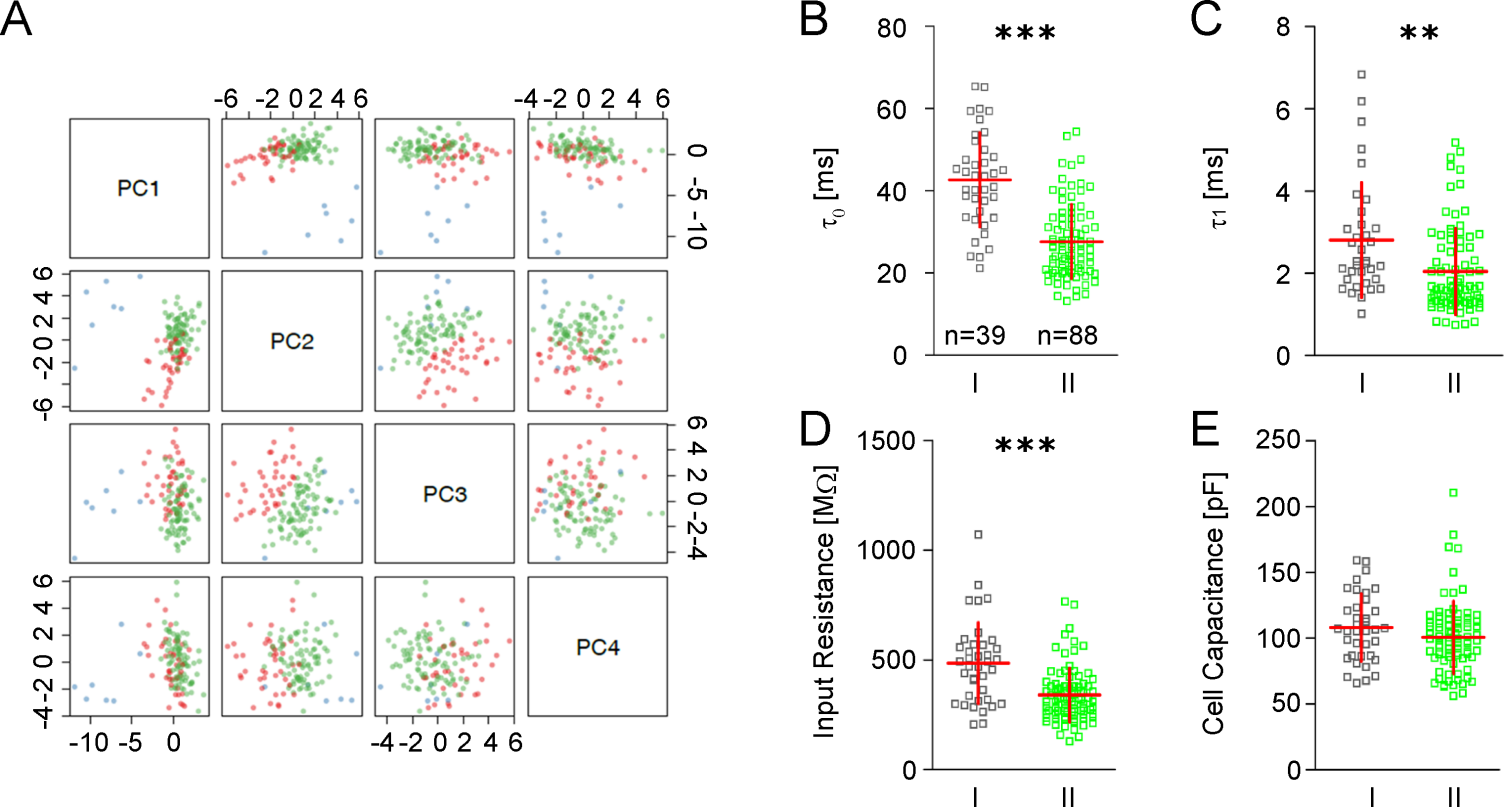
Principal component analysis and passive membrane properties in GIN. **A** Principal component analysis of 31 electrophysiological and 18 morphological parameters derived from 127 GIN (red and green dots) and 6 pyramidal cells (blue dots). Principal components PC1-PC4 are plotted pairwise. Red dots indicate group I GIN, green dots group II GIN. **B-E** Scatter plots of passive membrane properties of both GIN subgroups (horizontal red bar: mean value, vertical red bar: SD). **B** Somatic (τ_0_) and **C** dendritic (τ_1_) time constants; **D** input resistance (R_N_) and **E** cell capacitance (C_N_).

In an effort to identify possible GIN subgroups, we performed a principal component analysis (PCA) and k-means clustering based on a data set consisting of 31 electrophysiological and 18 morphological parameters derived from 127 GIN and 6 pyramidal cells. PC analysis proved to be a very reliable tool to separate GIN from pyramidal cells (Fig 1A, blue dots). The subsequently performed k-means clustering resulted in the prediction of three neuronal subgroups: one group of pyramidal cells and two distinct subgroups of GIN (Fig 1A, red and green dots, group I GIN [n = 39] and group II GIN [n = 88]). Post-analysis of the two identified GIN clusters revealed (1) that all GIN which expressed CB but not CR (n = 6) belonged to group I, (2) that 13 out of 14 GIN expressing NPY belonged to group II and, (3) that cells expressing CR were equally represented in both clusters. These observations indicate strong correlations between the neurochemical, electrophysiological and morphological properties of GIN of the cingulate cortex.

After the establishment of the two GIN subgroups, we performed statistical comparisons of single electrophysiological and morphological parameters in order to determine the specific differences between the clusters.

### Passive membrane properties

First, we compared the passive membrane properties of GIN of the two subgroups, i.e. (1) the somatic and (2) dendritic membrane time constant (τ_0_, τ_1_), (3) the input resistance (R_N_), and (4) the apparent cell capacitance (C_N_). Group I GIN exhibited significantly longer somatic and dendritic time constants (τ_0_: 42.7 ± 11.5 ms vs. 27.6 +/- 9.0 ms, *p*<0.0001, τ_1_: 2.8 ± 1.4 ms vs. 2.05 ± 1.0 ms; *p*<0.01; Fig 1B and 1C) and a significantly larger input resistance (486 ± 184 MΩ vs. 341 ± 122 MΩ; *p*<0.0001; Fig 1D). The cell capacitance was similar in both groups (108 ± 25.6 pF vs. 101 ± 27.4 pF; *p* = 0.17; Fig 1E).

### Current-voltage relationship

**Fig 2:**
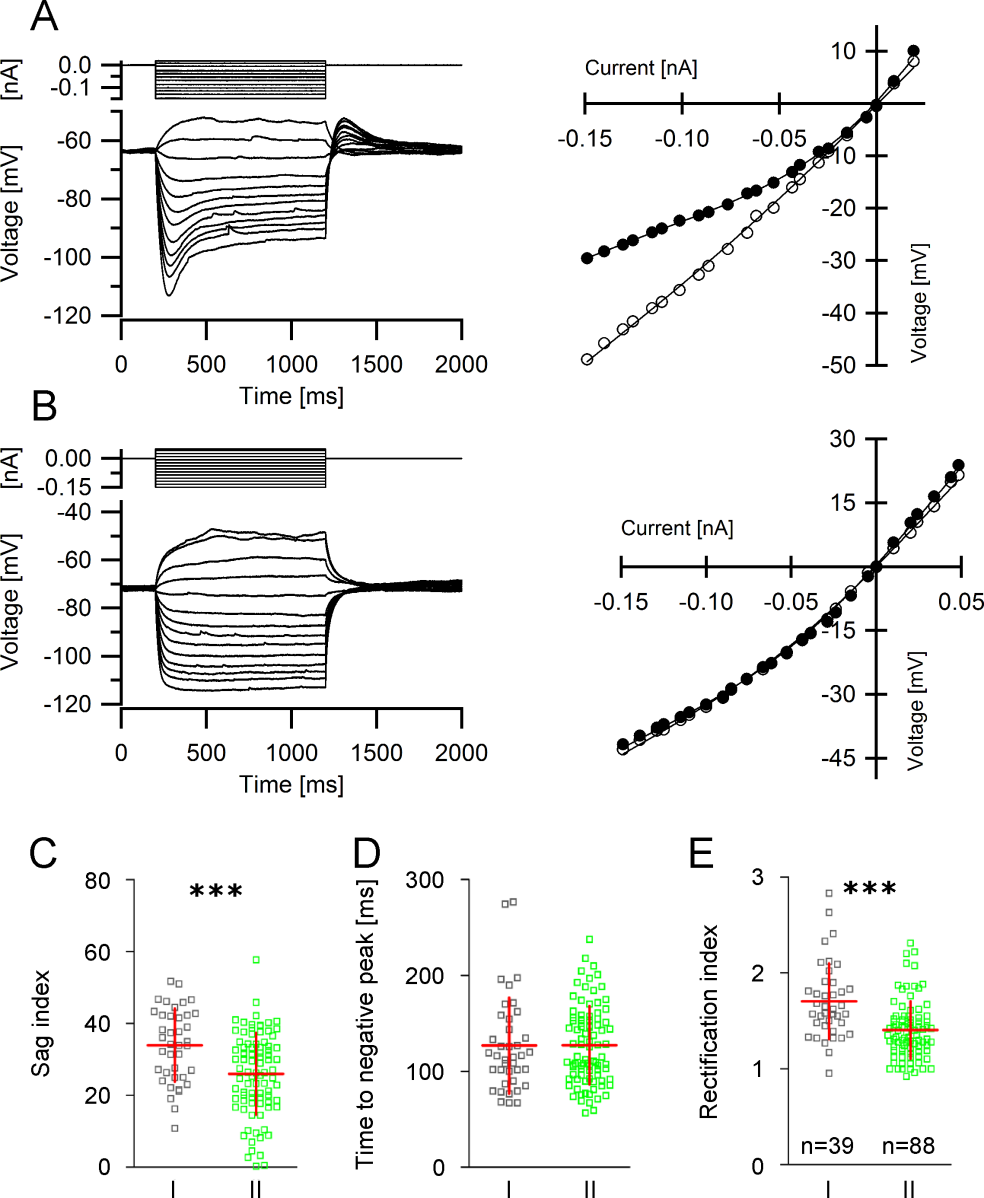
Current-voltage relationship in GIN. **A**, **B** *Left panels* Determination of the current-voltage-curve in GIN. A series of de- and hyperpolarizing current steps (upper traces) were injected into the cells and the corresponding voltage responses (lower traces) were measured. The subsequently obtained recordings are shown superimposed. Note the large sag potential in A. *Right panels* Corresponding *I-V* curves derived from the recordings shown in A and B. Closed circles depict the IV-relationship at steady state (i.e. at the end of the current pulse) and open circles show the IV-relationship at the time point of occurrence of the maximum negative peak potential. The difference between these two curves corresponds to the sag potential. **C-E** Differences in the IV-relationship in group I and group II GIN. Scatter plots of sag index (**C**), time to negative peak (**D**) and rectification index (**E**) in both GIN subgroups.

A special feature of GIN is the time-dependent inward rectification which becomes evident following the application of hyperpolarizing current pulses [19,20]. The great majority of GIN exhibited pronounced sag potentials (Fig 2A). In fact, only 20 out of 132 GIN displayed a sag potential smaller than 5 mV (Fig 2B). The degree of inward rectification was significantly larger in group I GIN compared to group II. Specifically, group I GIN displayed a significantly greater sag index (33.9 ± 10.2 vs. 26.0 ± 11.4; p<0.001, Fig 2C). Interestingly though, we found no differences in the time to the negative membrane potential peak following application of a hyperpolarizing current pulse (126.8 ± 49.9 ms vs. 127.2 ± 40.9 ms, *p* = 0.63, Mann-Whitney test, Fig 2D) indicating that HCN channel isoforms are equally represented in both GIN subgroups. In line with a significantly larger sag index, group I GIN also exhibited a larger rectification index (1.7 ± 039. vs. 1.4 ± 0.30, *p*<0.0001, Fig 2E).

### Properties of single action potentials

**Fig 3:**
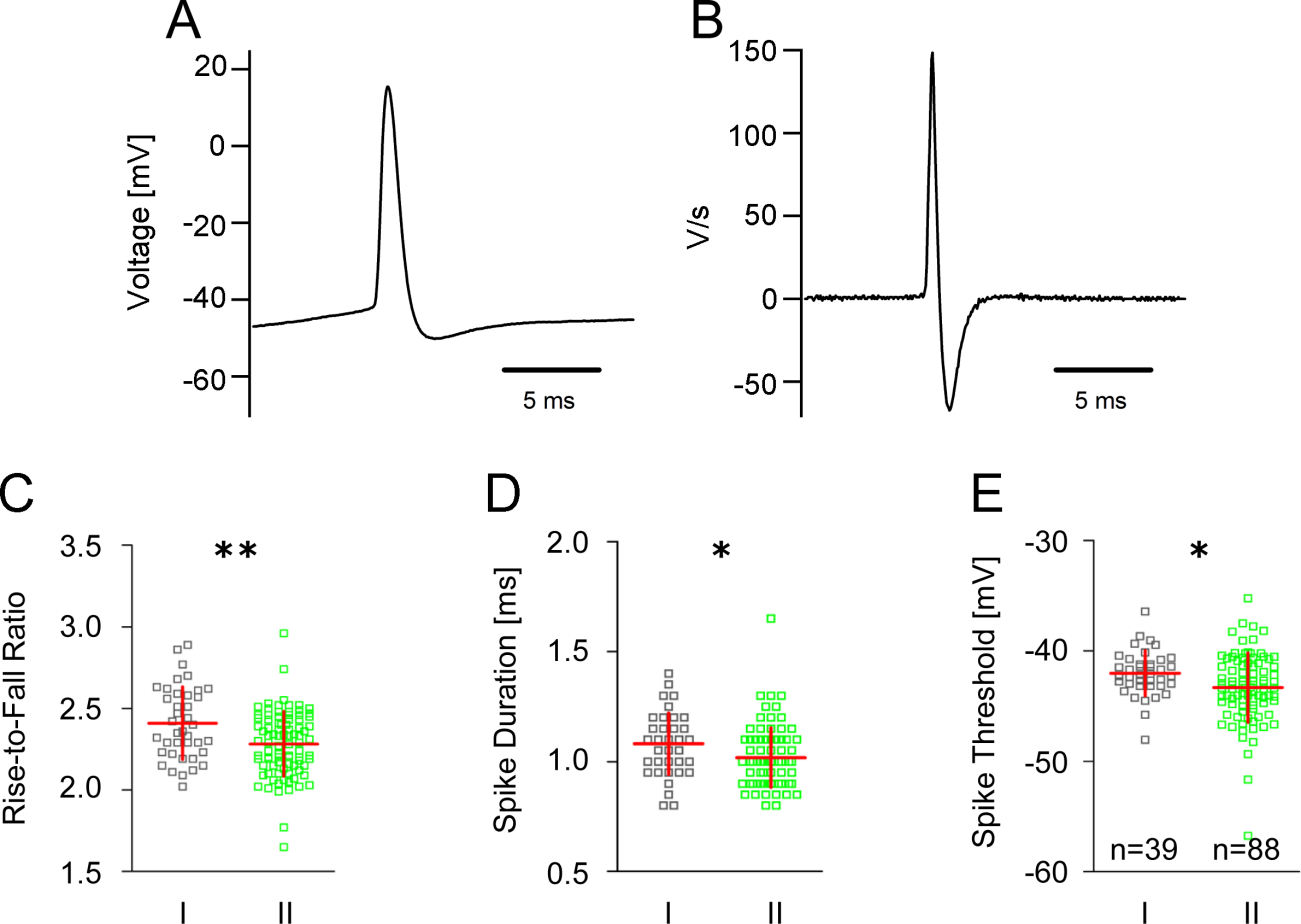
Single action potential kinetics in GIN. **A** Representative recording of a single action potential in GIN. **B** First derivative of the action potential shown in (A). **C-E** Scatter plots with mean and SD values in group I and group II GIN showing differences in the action potential rise-to-fall ratio (**C**), spike duration (**D**) and spike threshold (**E**).

Analysis of single spikes evoked with just suprathreshold intensities (Fig 3A) revealed marked differences between the spike kinetics of group I and group II GIN. The rise-to-fall ratio (determined from the first derivative of the spike, Fig 3B) in group I GIN was significantly larger compared to group II GIN (2.41 ± 0.22 vs. 2.28 ± 0.20, *p*<0.01; Fig 3C). Moreover, the spike duration of group II GIN was significantly shorter compared to group I GIN (1.02 ± 0.14 ms vs. 1.08 ± 0.14 ms, *p*<0.05, Fig 3D). In addition, analysis of the spike threshold revealed that group I GIN exhibited a significantly greater action potential threshold compared to group II GIN (-42.0 ± 2.1 mV vs. -43.3 ± 3.1 mV, *p*<0.05, Fig 3E). Single action potential amplitudes and spike rising slopes were similar in both subgroups of GIN (S1 Table).

### Patterns of evoked spike trains

**Fig 4:**
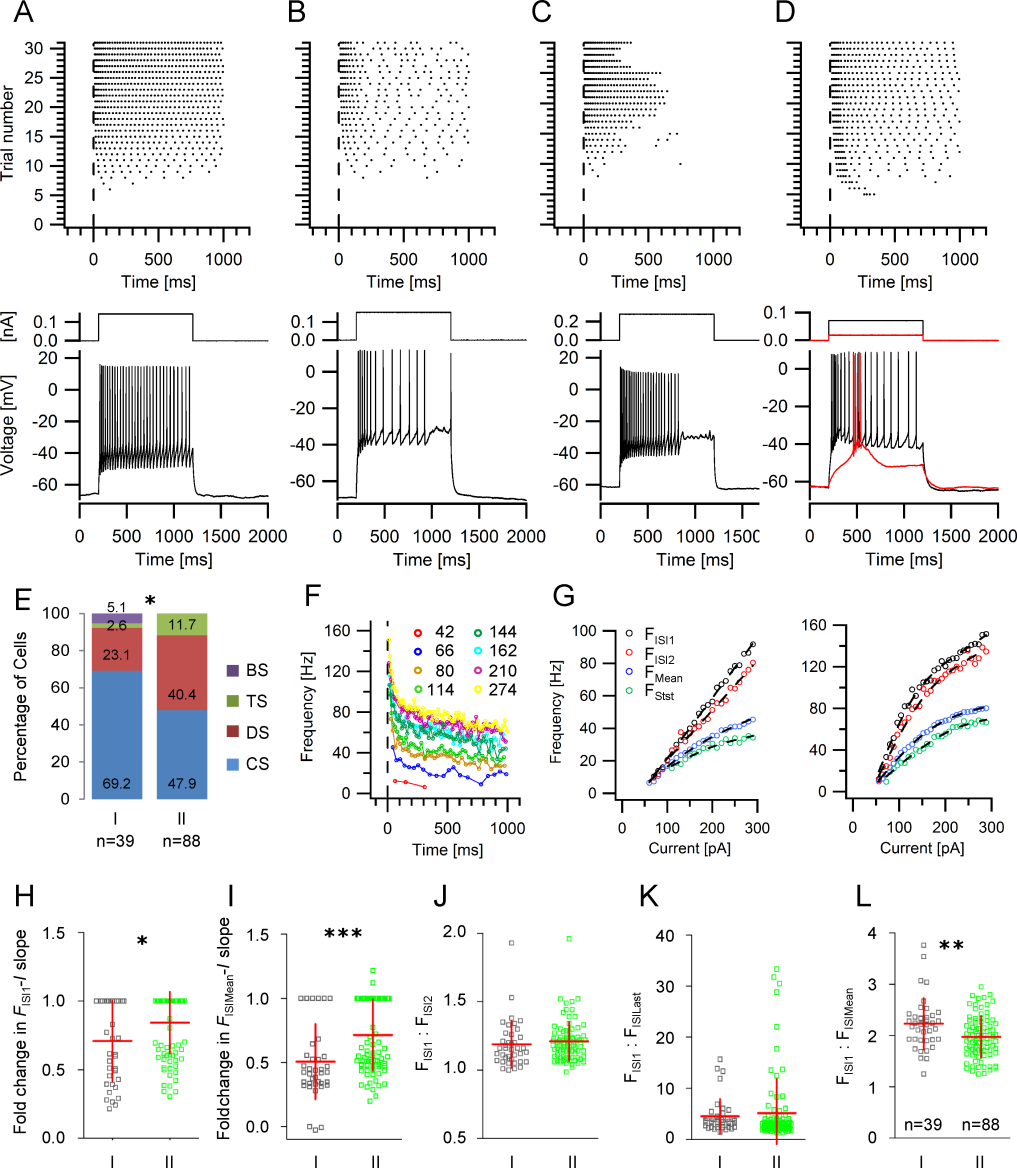
Action potential discharge properties in GIN. **A**-**D** Firing patterns in GIN. *Upper panels*: Representative raster plots of the different action potential discharge patterns in GIN. Each dot represents an action potential and each row of dots represents the response to a rectangular depolarizing current pulse (1 s). The current strengths increased from bottom to top. *Lower panels*: examples for spike trains evoked by medium current strengths from the raster plots above. **A** continuous spiking (CS), **B** discontinuous spiking (DS), **C** transient spiking (TS), **D** burst spiking (BS). **E** -Relative proportion of the different discharge patterns within the various GIN groups. **F** Representative frequency-time *(F-t)* relationship obtained from a continuously firing GIN. Current intensities are indicated. **G** Representative frequency-current (*F-I*) plots observed in GIN. Each diagram shows the *F-I* relationship for the first (black circles) and the second ISI (red circles), the steady state (last 300 ms, green circles) and the mean frequency (blue circles). Some GIN displayed a linear *F-I* relationship (*left panel*), while others exhibited a non-linear *F-I* relationship at the initial frequency (F_ISI1_, *right panel*). **H, I** Scatter plots displaying the relative change of the *F*_ISI1_-*I* (**H**) and *F*_ISIMean_-*I* (**I**) slopes. **J** Scatter plot of the ratio of the frequencies of the first and the second ISI in group I and II GIN. **K** Scatter plot of the ratio of the frequencies of the first and the last ISI in group I and II GIN. **L** Scatter plot of the ratio of the frequencies of the first and the mean frequency in group I and II GIN.

Phenomenologically, we distinguished four different spike train patterns in GIN (Fig 4A–4D): (1) spike trains without interrupts throughout the steps at medium and high current strength (continuously spiking, CS); this group also contains neurons with spike train adaptation (Fig 4A), (2) spike trains with clear and longer lasting interrupts during the current steps, even at medium and higher current strengths (discontinuously spiking, DS, Fig 4B), (3) spike trains with strong accommodation leading to the complete stop of spike generation during ongoing stimulation (transiently spiking, TS, Fig 4C), and (4) spike trains which, at any suprathreshold current strength, started with a high frequency discharge of a few action potentials superimposed on a low-threshold spike (burst spiking with all-or-nothing characteristics, BS, Fig 4D). Group I GIN had a significantly higher probability to exhibit a continuous discharge pattern compared to group II (69.2% vs. 47.9%, Mann-Whitney test, *p*<0.05; Fig 4E). Moreover, burst-spiking GIN were only found in group I GIN.

Next, we generated frequency-time-histograms (*F*-*t*-histograms) at different current intensities by plotting the reciprocal of each interspike interval (ISI) as a function of the corresponding spike time (e.g.: Fig 4F). The frequencies calculated from the first ISI (F_ISI1_), the second ISI (F_ISI2_), the mean frequency of the last 300 ms (steady-state frequency; F_stst_) and the mean frequency (F_Mean_) were then plotted as a function of the injected current (i.e. *F*-*I*-curve; Fig 4G, left and right panel). We found that group II GIN displayed a higher probability to exhibit a linear *F-I*-curve. Specifically, the relative slope change of the *F*_ISI1_-*I* plot was 0.71 (± 0.29) in group I GIN and 0.84 (± 0.22) in group II (*p*<0.05; Mann Whitney test; Fig 4H). In addition, the relative slope change of the *F*_ISIMean_-*I* plot was also significantly larger in group II GIN compared to group I (0.51 ± 0.29 vs. 0.71 ± 0.28; *p* = 0.0001, Fig 4I).

Frequency adaptation was calculated by determining the ratio of F_ISI1_ and F_ISI2_ (Fig 4J), the ratio of F_ISI1_ and of the frequency obtained from the last ISI (F_ISILast_, i.e. adaptation index, Fig 4K) and the ratio of F_ISI1_ and the average discharge frequency obtained from all ISIs (F_ISIMean_, Fig 4L). No statistically significant differences regarding the average F_ISI1_/F_ISI2_ and F_ISI1_/F_ISILast_ ratio could be detected among the two GIN subgroups (Fig 4J and 4K). However, we found a significantly higher F_ISI1_ to F_ISIMean_ ratio in group I GIN (2.23 ± 0.5, vs. 1.97 ± 0.41; *p*<0.01, Fig 4L).

### Changes in single spike kinetics during a train of action potentials

**Fig 5:**
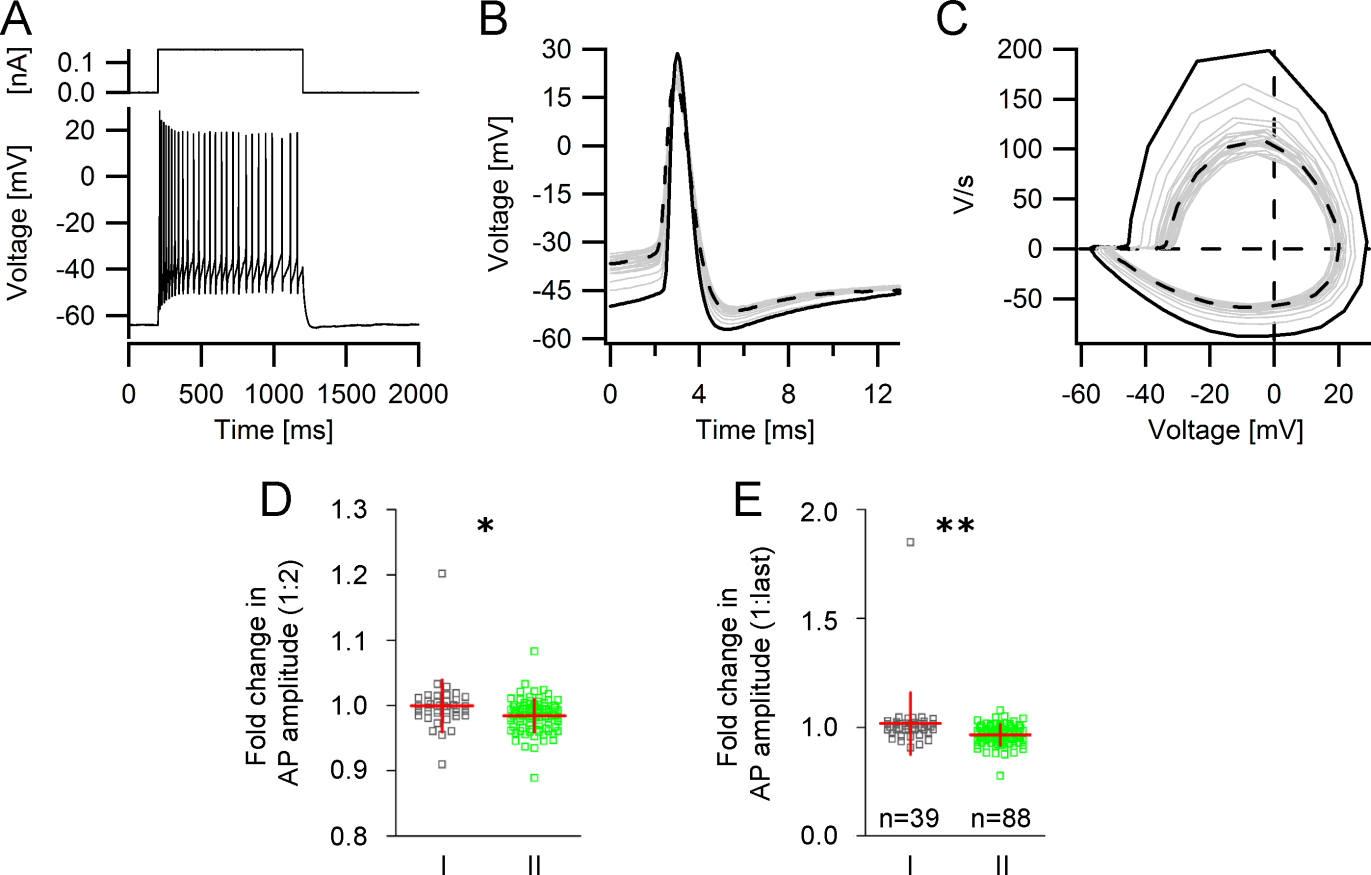
Changes in action potential kinetics during a train of action potentials. **A** Representative single trace of a repetitive spike discharge elicited by a depolarizing current step. **B** Single action potentials from (A) superimposed and aligned with respect to the peak amplitude. Black: first action potential in the train, grey: intermediate action potentials in the train. Black dashed line: last action potential in the train. **C** Phase plane plots of action potentials from (A) superimposed. Black: first action potential, grey: intermediate action potentials, black dashed line: last action potential of train. **D, E** Scatter plots showing that the degree of action potential shunting of the second (**D)** and last spike (**E**) was significantly greater in group II GIN.

The properties of single action potentials may change considerably during a spike train. An example of a spike train and the corresponding single action potentials, differentiated action potentials and phase plane plots can be seen in Fig 5A–5C. We compared the threshold, the amplitude, the rising slope, and the duration of the second and the last spike to the same parameters of the first spike of a spike train. The mean relative increase in spike threshold of the second spike, the relative change in spike duration of the second spike and the relative decline in the spike rising slope of the second spike were similar in both GIN subgroups (S1 Table). However, group II GIN displayed a greater reduction in the relative action potential amplitude of the second and of the last spike compared to the first spike. Specifically, the relative change in action potential amplitude of the second spike in group I GIN was 1.00 (± 0.040) and 0.98 (± 0.025) in group II GIN (*p*<0.05; Fig 5D). Similar to the relative change of the second spike amplitude compared to the first, we also observed a significant difference in the relative change of the last spike amplitude compared to the first in group I and II GIN. In group I GIN, the spike amplitude was relatively constant in a train of spikes and the fold change of the last spike amplitude compared to the first was 1.02 (± 0.14); in group II GIN, the relative change of the last spike amplitude compared to the first was significantly lower (0.97 ± 0.05; *p*<0.01, Fig 5E).

### Properties of spike afterhyperpolarization in GIN

**Fig 6:**
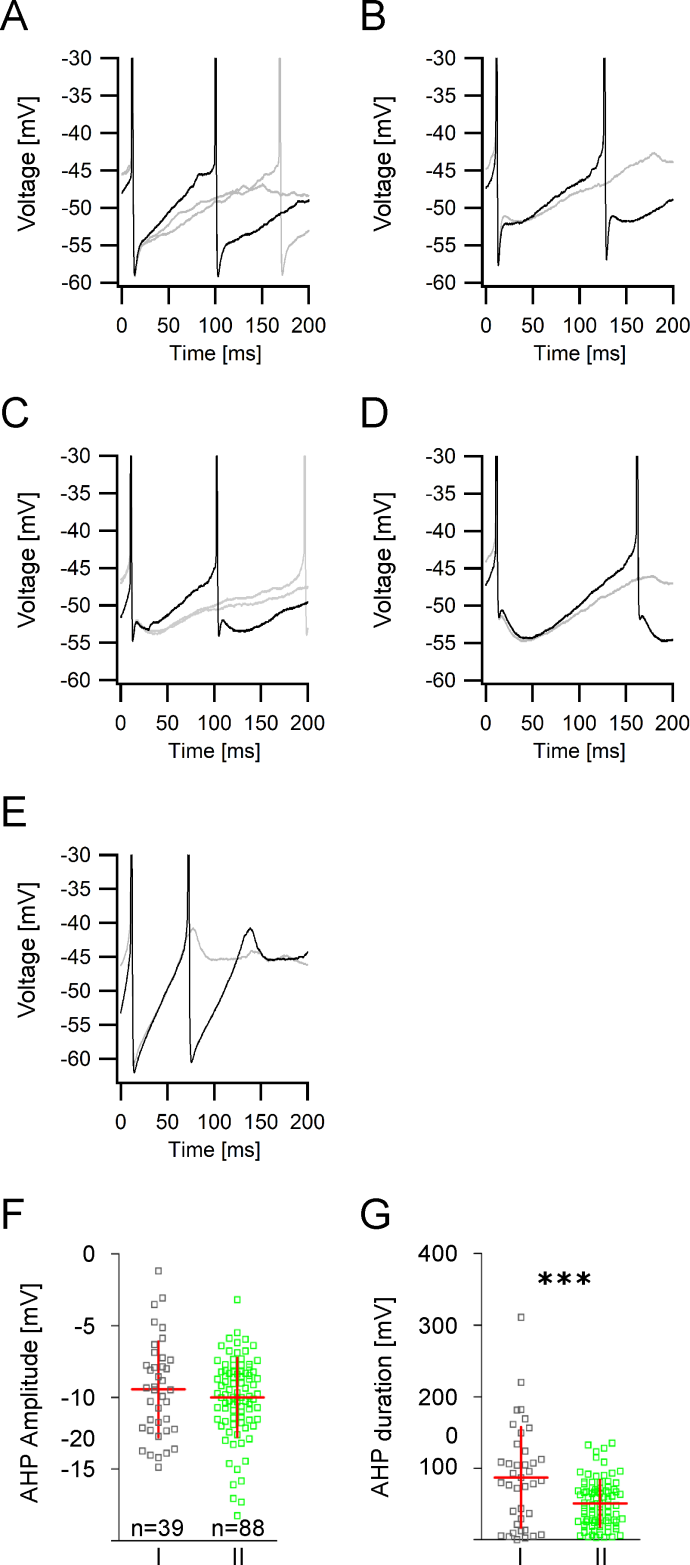
Action potential afterhyperpolarization properties in GIN. **A**-**E** Examples of different action potential afterhyperpolarizations (AHP) recorded in GIN following injections of just suprathreshold currents. Black: first AHP of a spike train. Grey: consecutive AHPs. **A** Fast AHP with a steep rising slope. **B** Fast initial AHP followed by a smaller slower AHP. **C** Representative AHP in which the fast initial AHP is comparable to the size of the slow AHP. **D** Very pronounced slow AHP component with only a small fast initial AHP. **E** Monophasic AHP. **F** Scatter plot showing the AHP amplitudes in group I and group II GIN. **G** Scatter plot showing that AHP durations in group II GIN are significantly shorter compared to group I GIN.

Interneurons have often been distinguished according to the properties of the spike afterhyperpolarization (AHP). In GIN, the time courses and magnitudes of the spike afterhyperpolarizations varied to a large degree as can be seen from Fig 6A–6E. The differences in the AHP kinetics depended predominantly on the presence of a second slower AHP component impacting on the overall amplitude, slope and duration of the AHP. Sometimes the fast initial AHP was very pronounced (Fig 6A), in many cases it was followed by a smaller slower afterhyperpolarization (Fig 6B), the amplitude of which was sometimes comparable to the size of the fast AHP (Fig 6C). In some GIN however, the fast initial AHP could barely be detected and the slower AHP was dominant (Fig 6D). The mean AHP amplitude in both GIN subgroups was similar (-9.94 ± 3.3 mV vs. -10.00 ± 2.77 mV, *p* = 0.33; Fig 6F). However, the mean AHP duration was significantly shorter in group II compared to group I GIN (51.0 ± 32.6 ms vs. 87.3 ± 70.0 ms, *p* = 0.0001; Fig 6G). GIN subgroups did not differ in the time to the AHP peak and in the AHP slope (S1 Table).

### Spontaneous synaptic activity in GIN

**Fig 7:**
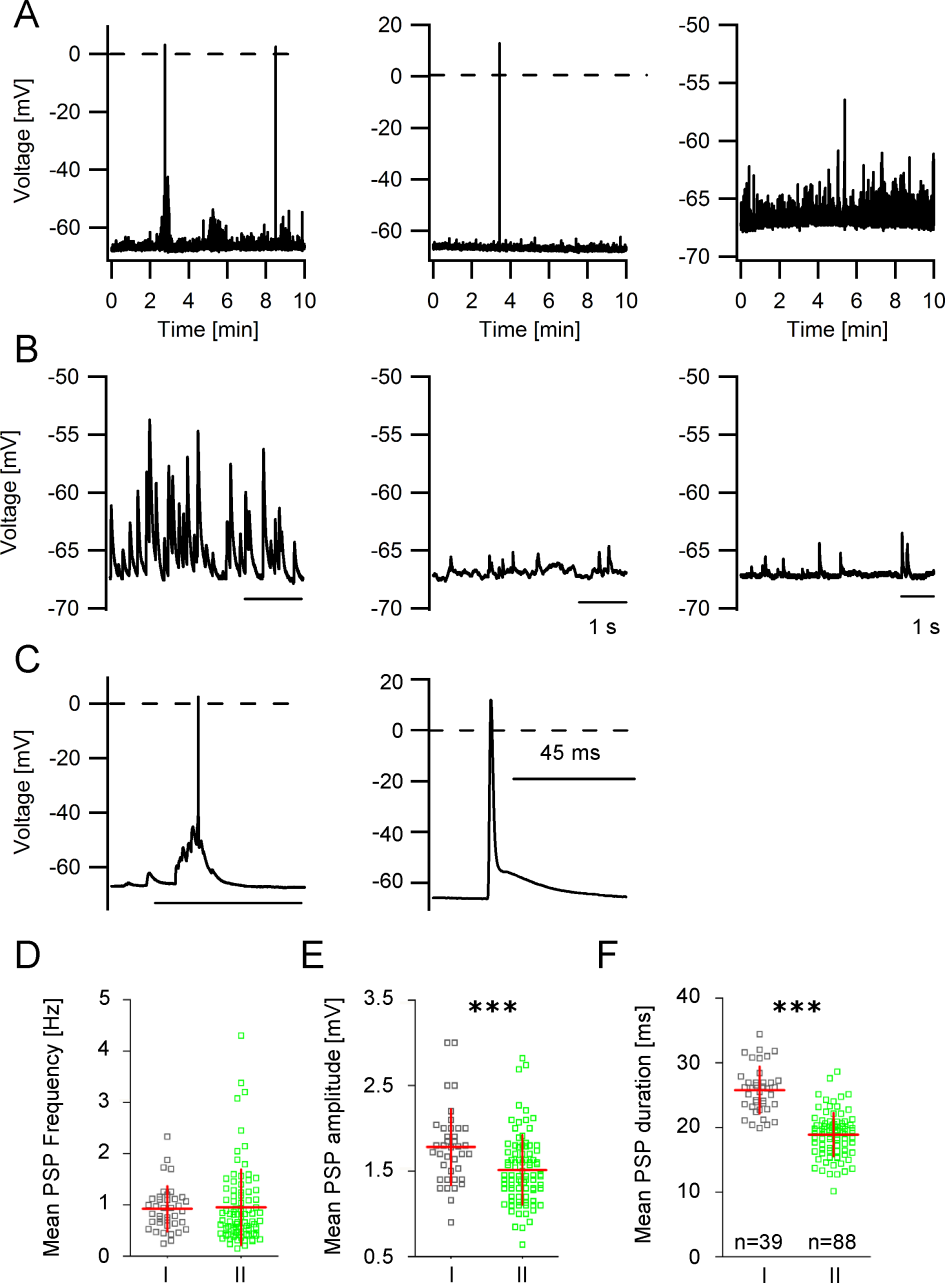
Properties of spontaneous postsynaptic potentials in GIN. **A** Representative recordings of spontaneous synaptic activity in different GIN. **B** Expanded traces of the voltage traces shown in A. **C** Expanded voltage traces (same recordings as in A) showing spontaneously induced action potentials. **D** PSP frequencies are similar in both GIN subgroups (scatter plots with mean values ± SD). **E** Scatter plot showing that PSP amplitudes and **F** PSP durations are significantly smaller in group II compared to group I GIN.

A unique feature of GIN in the cingulate cortex is the strong synaptic input which can be recorded as spontaneous events in the somata of the neurons. Fig 7A shows examples of voltage recordings (10 min in duration) in different GIN. Fig 7B displays parts of the same recordings on an expanded time scale and at higher amplitude resolution. Sometimes, the amplitudes of the synaptic potentials were large enough to trigger action potentials (Fig 7C). Analysis of these spontaneous postsynaptic potentials (PSPs) revealed that GIN received synaptic input at a mean frequency of around 1 Hz but no differences in the mean PSP frequency between group I and group II GIN could be detected (0.93 ± 0.43 Hz vs. 0.95 ± 0.74 Hz; *p* = 0.83; Fig 7D). PSP amplitude and duration were significantly smaller in group II GIN compared to group I (Fig 7D and 7E). In group I GIN, the mean PSP amplitude amounted to 1.78 ± 0.44 mV, in group II GIN the mean PSP amplitude was 1.5 ± 0.41 mV (*p*<0.001; Mann-Whitney test; Fig 7E). The PSP duration in group I GIN was 25.8 ± 3.6 ms and in group II GIN it was 18.9 ± 3.4 ms (*p*<0.0001; Fig 7F). In addition, analysis of the PSP slope revealed no differences between both types of GIN (S1 Table). Interestingly, spontaneous spikes (Fig 7C, right panel) which sometimes looked like axonal spikes [28] could be observed in 38 out of 132 GIN and in both GIN subtypes.

### Morphological classification of GIN subtypes

**Fig 8:**
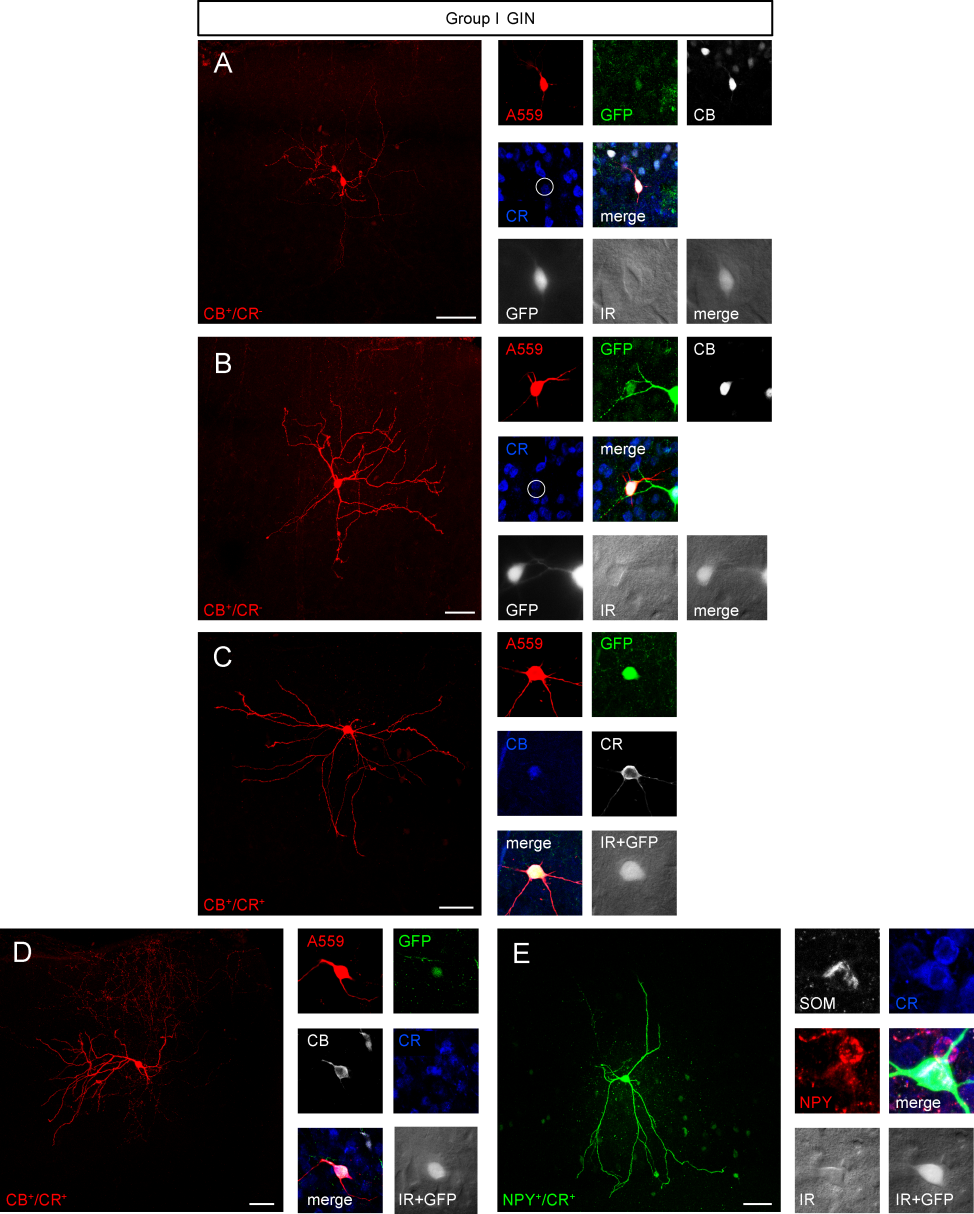
Morphological variety of group I GIN. **A**-**E**, *left panel* Confocal z-stack images as maximum intensity projections of representative group I GIN. Scalebars: 50 μm. *Right panel* Immunolabeling of biocytin-injected cells for GFP (green), CB (white or blue), CR (white or blue), SOM (white) or NPY (red). Note the lack of CB expression of the GIN shown in *A* and *B* (white open circle). Fluorescence (white, GFP) and infrared (IR)-DIC (grey) images of recorded cells were acquired prior recording.

**Fig 9:**
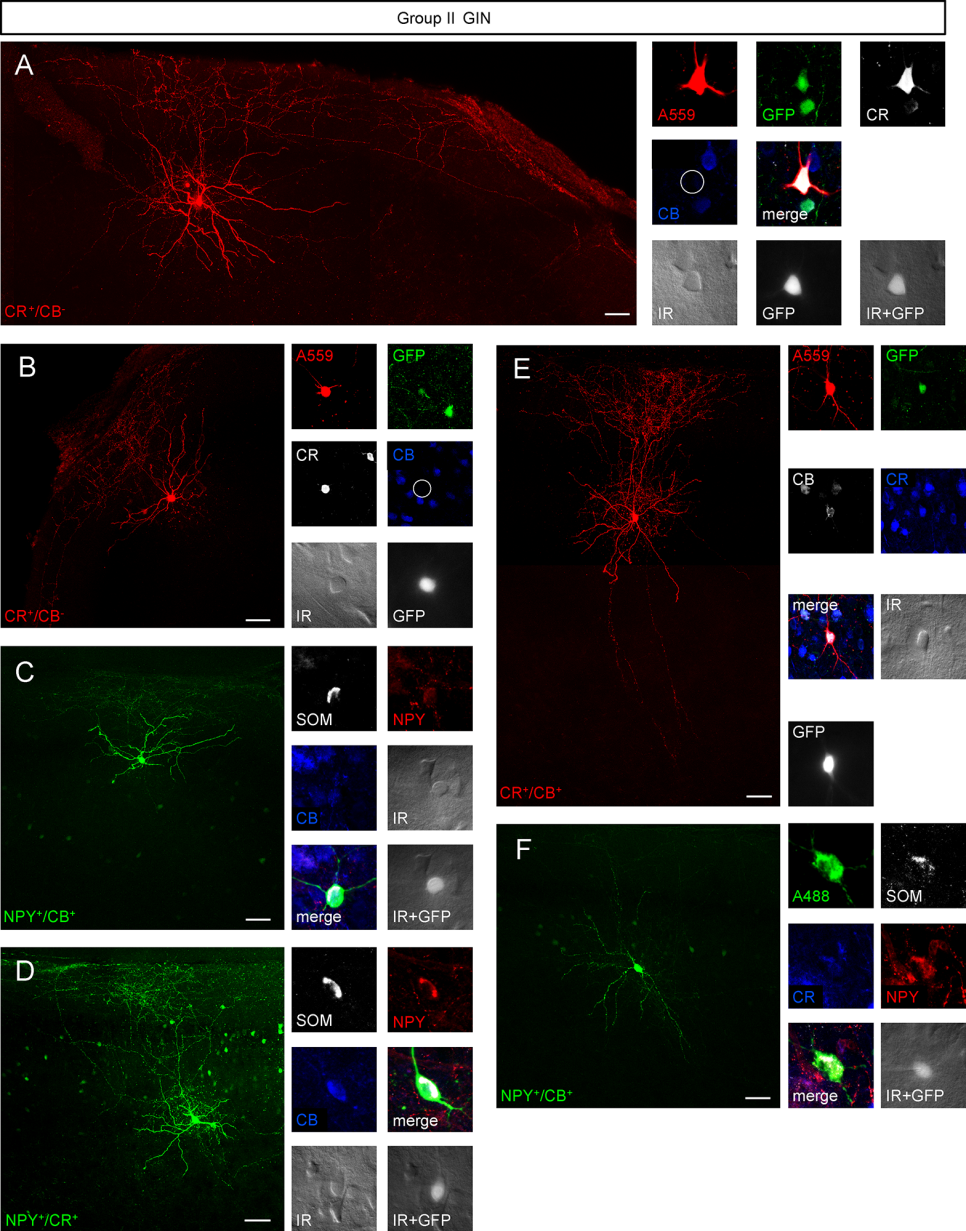
Morphological varieties in group II GIN. Many group I GIN classified as Martinotti cells with massive axonal arborizations in layer 1 and in the home layer. All scalebars: 50 μm. **A-F**, *left panel* Confocal Z-stack images of biocytin-injected GIN as maximum intensity projections. *Right panel* Corresponding immunolabelings of the cells shown in the *left panel*. Cells were labeled for GFP (green), CR (white or blue), CB (blue or white), SOM (white) or NPY (red). Fluorescence (GFP, white) and infrared-DIC (grey) images were acquired of cells in A-F prior recording.

**Fig 10:**
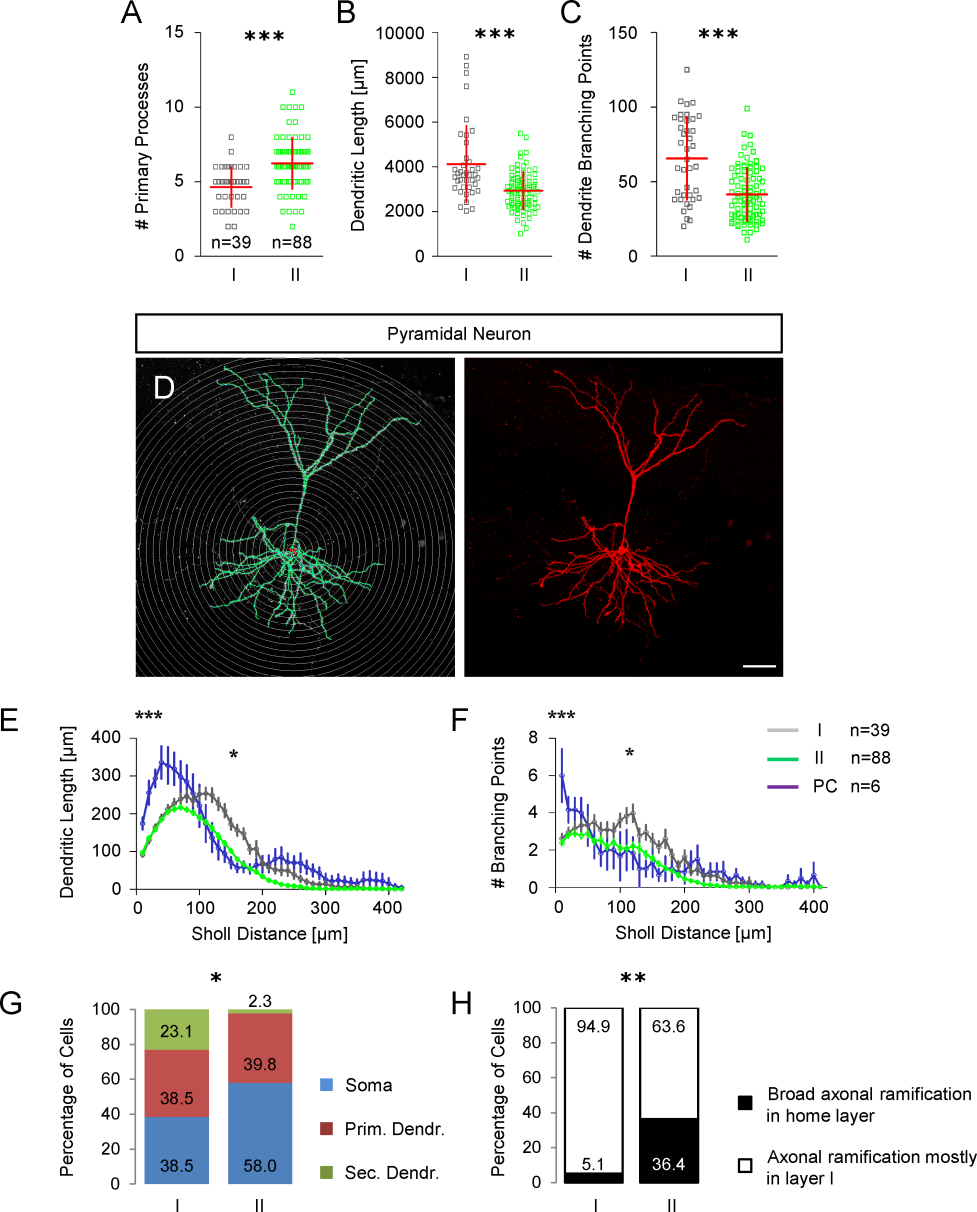
Morphological characterization of GIN in the cingulate cortex. **A** Scatter plot showing the number of primary processes in group I and group II GIN. **B** Scatter plot showing the total dendritic length in group I and group II GIN. **C** Scatter plot showing the total number of dendrite branching points in group I and group II GIN. **D** Representative morphology of a biocytin-injected pyramidal cell in the mouse cingulate cortex. Sholl sphere intervals: 10 μm; scalebar: 50 μm. Digital reconstruction in the *left panel*; maximum intensity projection of confocal Z-stack images of the corresponding pyramidal cell in the *right panel*. **E** Sholl analysis of dendrite length in group I (grey) and group II (green) GIN compared to pyramidal neurons (blue). The dendritic length is plotted as a function of the Sholl distance (mean ± SEM). Compared to GIN, we found a greater dendritic length in pyramidal neurons (two-way analysis of variance). Group I GIN vs. pyramidal neurons: 10 μm *p*<0.05; 20–50 μm *p*<0.001; 60 μm *p*<0.01; 110 μm *p*<0.05; 120–160 μm *p*<0.001; 170 μm *p*<0.01. Group II GIN vs. pyramidal neurons: 10 μm *p*<0.05; 20–60 μm *p*<0.001; 70 μm *p*<0.01; 80 μm *p*<0.05; 230 μm *p*<0.05. Group I GIN exhibited a greater dendritic complexity compared to group II GIN: 80 μm *p*<0.05; 100–190 μm *p*<0.001; 200–220 *p*<0.05; 230–240 μm *p*<0.01. **F** Branching points plotted as a function of the Sholl distance (mean ± SEM). Group I GIN vs. pyramidal neurons: 10 μm *p*<0.001; 120 μm *p*<0.05. Group II GIN vs. pyramidal neurons: 10 μm *p*<0.001. Group I GIN exhibited a greater dendritic complexity compared to Group II GIN: 60 μm *p*<0.01; 100–120 μm *p*<0.001; 140 μm p<0.001; 150–160 p<0.05; 170 *p*<0.001; 200 μm *p*<0.001; two-way analysis of variance. **G** Bar chart showing the relative percentages of different axon origins in both GIN subgroups. **H** Bar chart showing the relative percentages of axonal ramifications in the GIN home layer; comparison of group I vs. group II GIN.

Examples of biocytin-filled group I and group II GIN and pyramidal neurons can be seen in Figs 8–10. The distance of the labeled neurons (i.e. their somata) from the pia surface ranged between 30 and 440 μm, which corresponds to layer 1–3. The distance to the pial surface was similar in both GIN groups (S1 Table). CB^+^, CR-lacking GIN exclusively belonged to group I GIN (Fig 8A and 8B), whereas 13 out 14 NPY^+^ GIN belonged to group II GIN (Fig 9C, 9D and 9F). CR^+^ GIN were equally represented in both GIN subtypes (Fig 8C and 8D; Fig 9A, 9B, 9E and 9F).

### Somatodendritic variety of GIN

Analysis of the somatodendritic morphology in interneurons has been proposed a good discriminator for interneuron subtypes. Of all GIN analyzed, 41% displayed a multipolar somatodendritic morphology. Eight per cent of GIN had a tripolar morphology and 2% of GIN were bipolar. In 7% of GIN none of the above given groups fitted and we allocated these GIN to an 'other' group (S1 Fig). The remaining GIN were of the tufted type: 19% were single-, 12% bi-, and 11% modified single- or modified bi-tufted [29]. Subtle differences in the somatodendritic morphology between group I and group II GIN became evident: around one half of all group II GIN and only around one third of group I GIN exhibit a multipolar morphology (S1 Table). In turn nearly one half of group I GIN and less than one third of group II GIN are of the tufted type (48.72% vs. 27.27%, p<0.05, Mann-Whitney test, S1 Table). In addition, group I GIN had significantly fewer primary processes compared to group II GIN (4.6 ± 1.33 vs. 6.2 ± 1.7, *p*<0.0001; Fig 10A).

### Dendritic complexity of GIN

The total dendritic length in group I GIN was significantly longer compared to group II GIN (4119 ± 1711 μm vs. 2935 ± 836 μm, *p*<0.0001, Mann Whitney test; Fig 10B). Accordingly, the total number of branching points was significantly larger in group I GIN compared to group II (65.62 ± 27.8 vs. 41.42 ±17.7, *p*<0.0001; Mann Whitney test, Fig 10C). A Sholl analysis of the dendritic tree (Fig 10E) confirmed our findings above that group I GIN display a significantly higher dendritic complexity. Specifically, we found that the peripheral dendritic length (around 150 μm from soma) was significantly higher in group I GIN compared to group II. In addition, group I GIN had a higher number of branching points at around 100 μm Sholl distance (Fig 10F). Moreover, the Sholl analysis of dendritic length and branching points reliably separated GIN from pyramidal neurons. Analysis of the axon origin of GIN revealed that the probability of the axon originating from the soma was slightly smaller in group I GIN (38.5% vs. 59.0%; *p* = 0.075; Mann Whitney test; Fig 10G). In turn, the probability that the axon originated from a secondary dendrite was significantly higher in group I GIN (23.1% vs. 2.3%, *p*<0.05; Mann Whitney test; Fig 10G). Furthermore, group I GIN had a significantly lower probability of broad axonal ramifications in the home layer (5.1% vs. 36.4%, *p*<0.01, Mann Whitney test; Fig 10H).

### Dendritic spines in GIN

**Fig 11:**
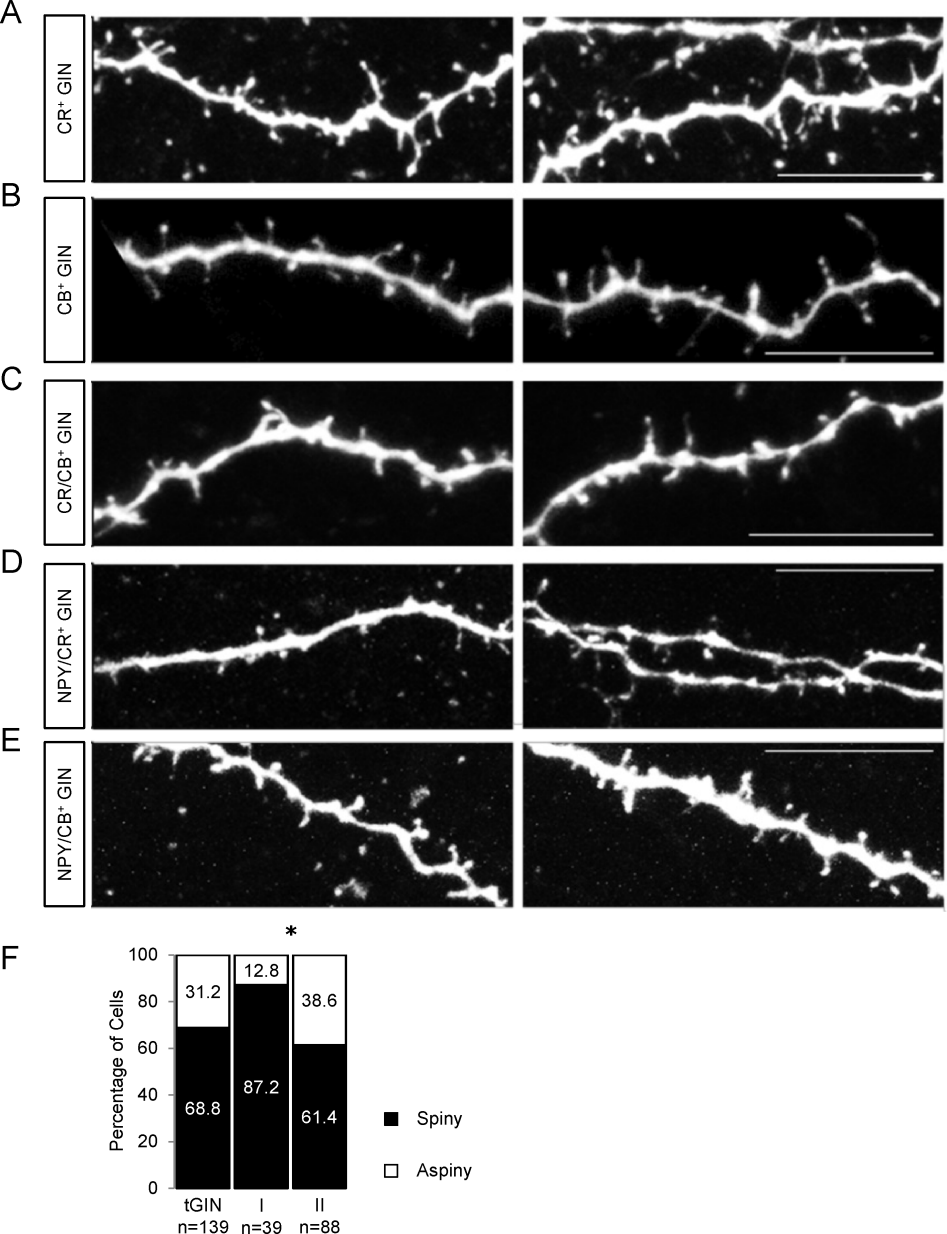
GIN of the cingulate cortex express dendritic spines. **A**-**E** Representative spiny dendrite morphologies of biocytin-injected GIN with different neurochemical phenotypes in the mouse cingulate cortex. Z-stack images were taken with a 63x objective on a laser scanning microscope; maximum intensity projections are shown here. Scalebars: 10 μm. **F** Bar chart exhibiting the relative portion of spiny (black) and aspiny (white) dendrites in all GIN (tGIN) and in both GIN subgroups.

We detected dendritic spines in around two thirds of all GIN. Such spines have previously been attributed to Martinotti cells [6, 30]. Examples of dendritic spines on different neurochemical GIN subtypes can be seen in Fig 11A–11E. The probability to express dendritic spines was significantly higher in group I GIN compared to group II (87.2% vs. 61.4%, *p*<0.05, Mann Whitney test, Fig 11F). This finding correlates with the observation that the highest probability to express dendritic spines can be found in CB^+^ GIN (5 out of 6 CB^+^ GIN), which exclusively belonged to group I. Moreover, the probability to express dendritic spines was lowest in NPY/CR-expressing GIN (2 out of 8 CR/NPY^+^ GIN) which almost exclusively belonged to group II (7 out of 8).

In summary, the existence of two putative subgroups of SOM^+^ GFP-expressing interneurons predicted by k-means clustering could be verified by the pairwise statistical analysis of single electrophysiological and morphological parameters of the neurons. Altogether, group I GIN have a higher input resistance than neurons of the group II, longer time constants and a stronger inward rectification in the higher membrane potential range. Neurons of the GIN I group display predominantly continuous action potential discharge patterns. The GIN II group comprises neurons which express NPY together with CB or CR, their total dendritic length is shorter, the number of dendritic branching points is smaller and the probability of dendritic spines is lower (see S1 Table).

## Discussion

The present study combined electrophysiological techniques, morphometric analysis and immunocytochemical methods to obtain detailed information on the properties of a GFP-labeled subgroup of somatostatin-expressing (SOM^+^) interneurons (GIN) of the cingulate cortex. Since all animals used in these experiments were older than 3 weeks (mean age: P31), we can preclude the occurrence of developmental and maturation-dependent differences in cell properties.

Multi-variate classification studies on cortical SOM^+^ interneurons reached the common conclusion that these neurons represent a remarkably heterogeneous group, and, in the somatosensory cortex, subclusters of these cells have been described [13,20,21,31,32]. In a recent study [2], we found neurochemically distinct subclusters of SOM^+^ interneurons in the agranular cingulate cortex. These clusters could be distinguished by their expression pattern of neuropeptides and calcium-binding proteins. In the study presented here, we focused on the question, whether the neurochemical subclusters of SOM^+^ interneurons in the cingulate cortex correlate with electrophysiological and/or morphological subgroups. Therefore, we collected electrophysiological and morphological data from GIN (n = 127) and pyramidal cells (n = 6) and subjected these data to a principal component analysis (PCA) and k-means clustering in order to predict possible clusters of GIN. About two thirds of all recorded neurons were, in addition, labeled immunocytochemically. A prerequisite for this study was that the neurochemical subgroups described previously were represented proportionally in the cell sample of the study presented here. Therefore, collection of electrophysiological and morphological data was terminated after having shown that we found similar proportions of neurochemical subtypes of GIN as previously reported [2], indicating that we obtained a representative sample size of all SOM^+^ interneurons in the cingulate cortex.

Our aim was to include as many parameters as possible into the multi-variate analysis in order to circumvent the frequent problem that different classification schemes are based on different distinguishing features. We, therefore, also included parameters that have been described as poor indicators of group membership in the past [21]. Limiting the number of parameters included in the PCA, which requires the selection of significant cell features, would have likely produced more clusters [33]. However, 'selection of cell features' is no longer compatible with an unbiased PCA. The PCA robustly predicted two cell clusters within the whole cell sample: GIN and pyramidal cells. Subsequent k-means clustering predicted two putative subpopulations of GIN within our sample. In addition, no pyramidal cell was found within one of the two GIN clusters, confirming the validity of our clustering approach.

Previous classification studies reported the existence of at least three clusters of SOM^+^ interneurons in the superficial layers of the somatosensory cortex [20,31,32]. In the study presented here, we detected only two groups of SOM^+^ interneurons in the cingulate cortex. These diverging numbers of SOM^+^ clusters might be due to: (1) Age of animals; (2) Brain region; (3) Different parameters used for classification. The classification scheme of Halabisky et al. [20] was partially based on postsynaptic currents without consideration of neuron morphology. Studies by McGarry et al. [31] and Santana et al. [32], respectively, used electrophysiological and morphological parameters for classification of juvenile SOM^+^ interneurons, but no neurochemical data. In addition to the electrophysiological and morphological properties of SOM^+^ interneurons, we also considered their neurochemical phenotype. We were thus able to refine the classification criteria of SOM^+^ interneurons resulting in the observation of intriguing differences. We found that all SOM^+^ interneurons that were positive for CB but negative for CR (6 out of 6) were present in group I GIN, whereas all but one GIN expressing NPY (in combination with either CR or CB, 13 out of 14) were present in group II GIN. Interestingly, CR expression and CR/CB coexpression were found to similar degrees in both groups of SOM^+^ interneurons. In order to fully understand the functional role of both SOM^+^ groups presented here, the role of both calcium-binding proteins and of NPY needs to be elucidated in these cells.

### Action potential discharge patterns in GIN

Analysis of the spiking pattern revealed two findings: 1) group I GIN display a greater probability of a continuous spiking pattern and 2) burst spiking GIN are only found in group I. These qualitative differences in spiking patterns could be quantified by comparing the degree of frequency adaptation between group I and group II GIN but also by determining the *F-I* relationship in both clusters. In general, SOM^+^ interneurons of the cingulate cortex seem to display a larger repertoire of discharge patterns compared to the somatosensory cortex, where most SOM^+^ interneurons display a regular action potential discharge [6,7,13,20,31,34–37]. Noteworthy, discontinuous spike discharges have also been reported in SOM^+^ interneurons in the lateral amygdala [38]. It is assumed that the properties of individual neurons are adapted to meet the demands of a given neuronal network. It is interesting in this respect that the cingulate cortex and the amygdala are both part of the limbic system and as such, do not strictly process sensory information but are rather important for coding the emotional content of a sensory stimulus. Intriguingly, painful stimuli trigger different neuronal activity patterns in the somatosensory and cingulate cortex [39,40].

### Properties of single action potentials group I and group II GIN

Analysis of single spikes in group I and group II GIN revealed differences in the spike kinetics. Group I GIN exhibit a greater rise-to-fall ratio and a slower spike. Both results indicate that the density of voltage-dependent potassium channels is lower in group I compared to group II GIN. The hypothesis that group I and group II GIN express a different density of potassium channels could further be substantiated by the finding that the degree of action potential shunting in a train of spikes was lower in group I compared to group II GIN. In line with this finding, we observed a longer AHP duration in group I GIN.

### Passive and active membrane parameters in group I and group II GIN

The passive membrane properties of GIN in the cingulate cortex are very similar to those of SOM^+^ interneurons in other cortical areas and brain regions [35,36]. Likewise, the time-dependent rectification in hyperpolarizing direction (as revealed by a strong sag potential) seems to be a classifying feature of SOM^+^ interneurons, independent of the brain area. Interestingly though, group I GIN exhibited a larger magnitude of inward rectification as well as a greater sag index, indicating that GIN are endowed with different densities of HCN channels. Since the kinetics of the sag potentials were similar in both GIN clusters, we assume that the HCN channel isoforms, are expressed in both GIN types to a similar degree.

### Morphological differences between group I and group II GIN

Analysis of the morphology of GIN in the cingulate cortex confirmed the hypothesis that the majority of layer 2/3 SOM^+^ interneurons presented here may be classified as Martinotti cells with a multipolar or tufted somatodendritic morphology (for review see [30]). In agreement with this finding, we observed dendritic spines in the majority of GIN. The probability to develop spines was highest in CB^+^/CR^-^ GIN (5 out of 6) that exclusively belonged to group I GIN.

To our surprise, some GIN had a basket cell like morphology. However, neither parvalbumin nor cholecystokinin expression has been observed in GIN of the cingulate cortex [2]. Most GIN with a basket cell like morphology belonged to group I. Moreover, group I GIN displayed a lower number of primary processes compared to group II GIN. Sholl analysis in turn revealed that type I GIN displayed a greater degree of dendritic branching in the peripheral Sholl distances. In agreement with this finding, we observed a shorter total dendritic length and a smaller dendrite branching number in type II GIN. Altogether, GIN of the cingulate cortex seem to have a more complex dendritic morphology compared to SOM^+^ interneurons of other brain regions. The total dendritic length in presubicular SOM^+^ interneurons [36] and in GIN of the somatosensory cortex [31] for example is shorter compared to that in GIN of the cingulate cortex. One possible explanation for this discrepancy could be animal age (juvenile vs. adult GIN, [31]) or brain region [36].

Analysis of the axons in group I and group II GIN revealed that the axons not only originated from the somata but to a similar degree also from primary or secondary dendrites. The probability that the axon originated from the secondary dendrite was higher in group I GIN, indicating not only differences in the dendritic but also axonal morphology of both groups. In addition, group II GIN were more likely to exhibit vast axonal arborizations in the home layer of a respective GIN, possibly indicating a greater percentage of Martinotti-type cells in group II GIN.

### Synaptic integration of group I and group II GIN

Given the fact that group I GIN exhibited a bigger fraction of basket-like cells, it is conceivable that these SOM^+^ interneurons exert a different synaptic output onto pyramidal neurons compared to group II GIN. Martinotti cells have been associated with disynaptic inhibition between pyramidal neurons [41,42] and have been proposed to function as regulators of neocortical activity and to gate synaptic plasticity [43]. Recently, Martinotti cells have been reported to project non-specifically to almost all neuronal types within the home layer or even to neuronal types across several layers [44]. It will be interesting to compare the synaptic output between group I and group II GIN onto neighboring cells in the future. Given the finding that the axonal tree seemed less elaborate in group I GIN, we propose that both subgroups provide different synaptic outputs onto their target neurons. Regarding the synaptic input, we found a greater PSP amplitude and duration in group I GIN. A likely reason for these differences could be the greater input resistance and membrane time constants in group I GIN. Another possibility could be that group II GIN are bestowed with a greater density of small-conductance (SK) potassium channels that have been shown to suppress EPSPs [45]. Interestingly, calretinin expression has been correlated with SK channel expression in the past [9] and preliminary evidence from our laboratory suggests that SK channels are indeed expressed in GIN and modulate single spike kinetics. It will be interesting to examine in the future, whether SK channel densities differ in these GIN subtypes. Preliminary data from our laboratory suggest a massive GABAergic input to GIN probably originating from VIP^+^ interneurons [46]. The source of synaptic input onto GIN however, was not identified in this study and it is likely that the two types of GIN presented here receive synaptic input from distinct sources [44].

## Conclusion

In summary, we propose that GIN of the cingulate cortex exhibit a very diverse but yet related group of cells that is composed of at least two distinct subpopulations: All GIN express somatostatin, are located in layers 2 and 3 and lack the expression of parvalbumin, VIP, CCK and bNOS [2]. The great majority of GIN display inward rectification and a pronounced sag potential. Moreover, most GIN receive strong excitatory and inhibitory synaptic input as revealed by the recordings of spontaneous synaptic activity. At times, we could observe synaptically evoked spontaneous action potentials but also axonal spikes without prior depolarization (see: Fig 7C). Interestingly, axonal spikes have been reported in PV^+^ fast-spiking interneurons of the hippocampus [47]. However, there are some cell properties which were observed in only a small fraction of the GIN population of the cingulate cortex (e.g. bursting discharge pattern, outward rectification or expression of CB without CR). Based on a variety of electrophysiological, neurochemical and morphological criteria, we identified two subgroups of GIN and we therefore propose that CB^+^ and CR-lacking SOM^+^ interneurons as well as NPY^+^ SOM^+^ interneurons represent functionally distinct classes of SOM^+^ interneurons in the adult mouse cingulate cortex. In order to identify further GIN subgroups, it seems necessary to find additional criteria, in particular those which reflect the functions of the neurons within their local circuit. Preliminary data from our laboratory suggest that all GIN of the cingulate cortex investigated so far receive excitatory synaptic input which displays certain types of short-term plasticity. We assume that the investigation of the function of GIN within the local circuits of the cingulate cortex will eventually lead to a functional classification of these cells and will give an answer to the intriguing question, why an interneuron expresses two neuropeptides as co-transmitters.

## Supporting information

S1 Fig Somatodendritic variety in SOM^+^ interneurons. (TIF)

S1 Table Summary of electrophysiological and morphological properties of Group I and Group II GIN. (DOCX)
