# Supplementary figures and images for: Two types of somatostatin-expressing GABAergic interneurons in the superficial layers of the mouse cingulate cortex
Source: PLoS One. 2018 Jul 12;13(7):e0200567. doi: 10.1371/journal.pone.0200567 (PMC6042774; doi:10.1371/journal.pone.0200567)

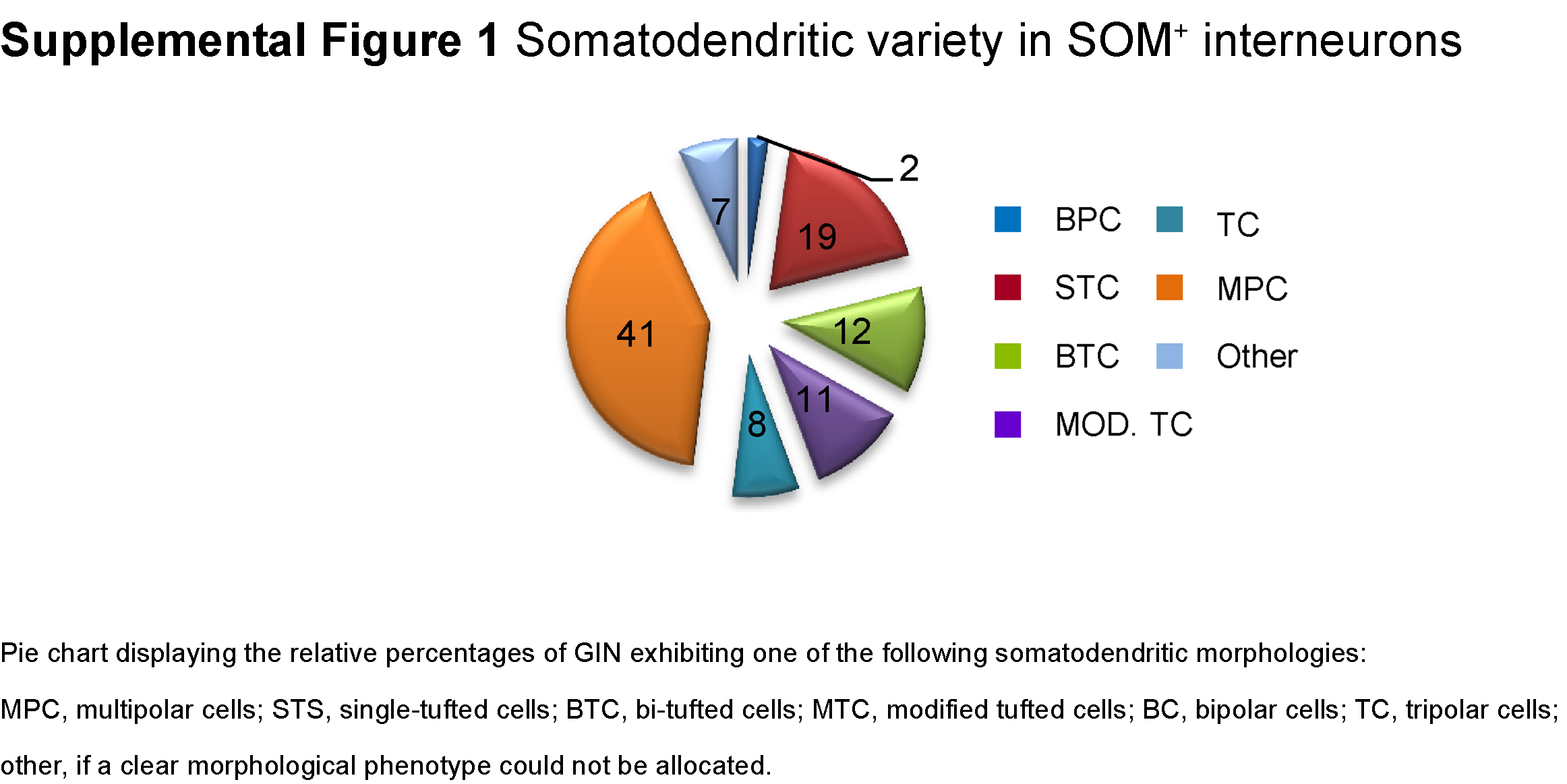

Supplement: S1 Fig — (TIF) [file pone.0200567.s001.tif]
