# Supplementary material for: Two types of somatostatin-expressing GABAergic interneurons in the superficial layers of the mouse cingulate cortex
Source: PLoS One. 2018 Jul 12;13(7):e0200567. doi: 10.1371/journal.pone.0200567 (PMC6042774; doi:10.1371/journal.pone.0200567)
Supplement: S1 Table — (DOCX) [file pone.0200567.s002.docx]

**Supplemental Data**

**Table 1: Summary of electrophysiological and morphological properties of Group I and Group II GIN**

| **Parameter** | **GIN I (CB^+^)** | **GIN II (NPY^+^)** | ***p*** |
| --- | --- | --- | --- |
| *n=39 n=88*  *Passive membrane properties* | | | |
| Capacitance [pF] | 108 ± 25.6 | 101 ± 27.4 | ns |
| τ_0_ [ms] | 42.7 ± 11.5 | 27.6 +/- 9.0 | <0.0001 |
| τ_1_ [ms] | 2.8 ± 1.4 | 2.05 ± 1.0 | <0.01 |
| Input Resistance [MΩ] | 486 ± 184 | 341 ± 122 | <0.0001 |
| *Current-VoltageRelationship* | | | |
| Sag Index | 33.9 ± 10.2 | 26.0 ± 11.4 | <0.01 |
| Time to negative Peak [ms] | 126.8 ± 49.9 | 127.2 ± 40.9 | ns |
| Rectification Index | 1.7 ± 039 | 1.4 ± 0.30 | <0.0001 |
| *Single Spike Properties* | | | |
| Rise-to-Fall Ratio | 2.41 ± 0.22 | 2.28 ± 0.20 | <0.01 |
| AP Duration [ms] | 1.08 ± 0.14 | 1.02 ± 0.14 | <0.05 |
| AP Rising Slope [V/s] | 145.1 ± 32.78 | 151.2 ± 28.05 | ns |
| APThreshold [mV] | -42.0 ± 2.1 | -43.3 ± 3.1 | <0.05 |
| *Action Potential Discharge Patterns* | | | |
| ContinuousDischarge Pattern [%] | 69.2 | 47.9 | <0.05 |
| DicontinuousDischarge Pattern [%] | 23.1 | 40.4 | ns |
| BurstingDicharge Pattern [%] | 5.1 | 0 | ns |
| Transient Discharge Pattern [%] | 2.6 | 11.7 | ns |
| Relative Slope Change of *F*_ISI1_-*I* Plot | 0.71 ± 0.29 | 0.84 ± 0.22 | <0.05 |
| Relative Slope Change of *F*_ISIMean_-*I* Plot | 0.51 ± 0.29 | 0.71 ± 0.28 | <0.0001 |
| F_ISI1_/F_ISI2_ | 1.19 ± 0.17 | 1.21 ± 0.14 | ns |
| F_ISI1_/F_ISILast_ | 4.53 ± 3.38 | 5.16 ± 6.64 | ns |
| F_ISI1_/F_ISIMean_ | 2.23 ± 0.5 | 1.97 ± 0.41 | <0.01 |
| *Relative Action Potential Changes in a Train of Spikes* | | | |
| Threshold AP2 : AP1 | 0.90 ± 0.03 | 0.90 ± 0.03 | ns |
| Threshold AP last : AP1 | 0.77 ± 0.06 | 0.79 ± 0.05 | ns |
| Amplitude AP2 : AP1 | 1.00 ± 0.040 | 0.98 ± 0.025 | <0.05 |
| Amplitude AP last : AP1 | 1.02 ± 0.14 | 0.97 ± 0.05 | <0.01 |
| Duration AP2 : AP1 | 1.08 ± 0.04 | 1.08 ± 0.04 | ns |
| Duration AP last : AP1 | 1.20 ± 0.10 | 1.18 ± 0.08 | ns |
| Rising Slope AP2 : AP1 | 0.80 ± 0.07 | 0.80 ± 0.07 | ns |
| Rising Slope AP last : AP1 | 0.62 ± 0.09 | 0.60 ±0.11 | ns |
| *Spike Afterhyperpolarization Properties* | | | |
| AHP Amplitude [mV] | -9.94 ± 3.3 | -10.00 ± 2.77 | ns |
| AHP Duration [ms] | 87.3 ± 70.0 | 51.0 ± 32.6 | =0.0001 |
| AHP Slope [V/s] | 0.65 ± 0.56 | 0.73 ± 0.43 | ns |
| AHP Time to Peak | 4.17 ± 1.80 | 3.80 ± 1.15 | ns |
| *Spontaneous synaptic Potentials* | | | |
| Mean PSP Frequency [Hz] | 0.93 ± 0.43 | 0.95 ± 0.74 | ns |
| Mean PSP Amplitude [mV] | 1.78 ± 0.44 | 1.5 ± 0.41 | <0.001 |
| Mean PSP Slope [V/s] | 0.39 ± 0.11 | 0.42 ± 0.13 | ns |
| Mean PSP Duration [ms] | 25.8 ± 3.6 | 18.9 ± 3.4 | <0.0001 |
| *Dendrite Properties* | | | |
| Number of primary processes | 4.6 ± 1.33 | 6.2 ± 1.7 | <0.0001 |
| Multipolar soma [%] | 33.33 | 51.14 | ns |
| Tufted soma [%] | 48.72 | 27.27 | <0.05 |
| Total dendritic Length [µm] | 4119 ± 1711 | 2935 ± 836 | <0.0001 |
| Total Number of branching Points | 65.62 ± 27.8 | 41.42 ± 17.7 | <0.0001 |
| Dendrite Length at 10 µm | 91.85 ± 38.38 | 97.86 ± 29.16 | ns |
| Dendrite Length at 100 µm | 252.22 ± 101.57 | 189.83 ± 65.75 | <0.001 |
| Dendrite Length at 200 µm | 69.50 ± 55.36 | 33.81 ± 45.73 | <0.05 |
| Dendrite Length at 300 µm | 13.81 ± 57.42 | 1.02 ± 4.93 | ns |
| Number of branching Points at 10 µm | 2.63 ± 1.66 | 2.39 ± 1.64 | ns |
| Number of branching Points at 100 µm | 3.60 ± 2.33 | 2.11 ± 1.97 | *p*<0.001 |
| Number of branching Points at 200 µm | 1.65 ± 2.20 | 0.45 ± 0.95 | *p*<0.001 |
| Number of branching Points at 300 µm | 0.30 ± 1.60 | 0.02 ± 0.14 | ns |
| Presence of Spines [%] | 87.2 | 61.4 | <0.05 |
| *Axon Properties* | | | |
| Axon origin soma [%] | 38.5 | 59.0 | ns |
| Axon origin primary dendrite [%] | 38.5 | 39.8 | ns |
| Axon origin secondary dendrite [%] | 23.1 | 2.3 | <0.05 |
| Axon Ramification in Home Layer [%] | 5.1 | 36.4 | <0.01 |
|  |  |  |  |
